# Inositol trisphosphate receptor-mediated Ca^2+^ signalling stimulates mitochondrial function and gene expression in core myopathy patients

**DOI:** 10.1093/hmg/ddy149

**Published:** 2018-04-25

**Authors:** Matteo Suman, Jenny A Sharpe, Robert B Bentham, Vassilios N Kotiadis, Michela Menegollo, Viviana Pignataro, Jordi Molgó, Francesco Muntoni, Michael R Duchen, Elena Pegoraro, Gyorgy Szabadkai

**Affiliations:** 1 Department of Biomedical Sciences, University of Padua, 35131 Padua, Italy; 2 Neuromuscular Unit, Department of Neuroscience, University of Padova, 35131 Padua, Italy; 3 Department of Cell and Developmental Biology, Consortium for Mitochondrial Research, University College London, London WC1E 6BT, UK; 4 The Francis Crick Institute, London NW1 1AT, UK; 5 Commissariat à l'Energie Atomique et aux Énergies Alternatives (CEA), Institut des Sciences du Vivant Frédéric Joliot, Université Paris-Saclay, Service d'Ingénierie Moléculaire des Protéines, Gif-sur-Yvette 91191, France; 6 Institut des Neurosciences Paris-Saclay, UMR 9197, Centre National de la Recherche Scientifique (CNRS)/Université Paris-Sud, Gif-sur-Yvette 91198, France; 7 University College London, Great Ormond Street Institute of Child Health Dubowitz Neuromuscular Centre and Medical Research Council (MRC) Centre for Neuromuscular Diseases, London WC1N 1EH, UK

## Abstract

Core myopathies are a group of childhood muscle disorders caused by mutations of the ryanodine receptor (RyR1), the Ca^2+^ release channel of the sarcoplasmic reticulum. These mutations have previously been associated with elevated inositol trisphosphate receptor (IP3R) levels in skeletal muscle myotubes derived from patients. However, the functional relevance and the relationship of IP3R mediated Ca^2+^ signalling with the pathophysiology of the disease is unclear. It has also been suggested that mitochondrial dysfunction underlies the development of central and diffuse multi-mini-cores, devoid of mitochondrial activity, which is a key pathological consequence of RyR1 mutations. Here we used muscle biopsies of central core and multi-minicore disease patients with RyR1 mutations, as well as cellular and *in vivo* mouse models of the disease to characterize global cellular and mitochondrial Ca^2+^ signalling, mitochondrial function and gene expression associated with the disease. We show that RyR1 mutations that lead to the depletion of the channel are associated with increased IP_3_-mediated nuclear and mitochondrial Ca^2+^ signals and increased mitochondrial activity. Moreover, western blot and microarray analysis indicated enhanced mitochondrial biogenesis at the transcriptional and protein levels and was reflected in increased mitochondrial DNA content. The phenotype was recapitulated by *RYR1* silencing in mouse cellular myotube models. Altogether, these data indicate that remodelling of skeletal muscle Ca^2+^ signalling following loss of functional RyR1 mediates bioenergetic adaptation.

## Introduction

Mutations of the type 1 ryanodine receptor (RyR1), a key sarcoplasmic reticulum (SR) Ca^2+^ release channel almost exclusively expressed in skeletal muscle, are one of the most common causes of non-dystrophic congenital myopathies. Different mode of inheritance of mutations in the *RYR1* gene and associated distinct clinico-pathological phenotypes are recognized: the dominantly inherited central core disease (CCD, MIM#11700); autosomal recessive multi-minicore disease (MmD, MIM #255320), RYR1-related centronuclear myopathy (CNM) (1) and congenital fibre-type disproportion (CFTD, MIM #255310) (2). In addition, association of malignant hyperthermia susceptibility (MHS, MIM# 145600) and exertional rhabdomyolysis (3) traits are also associated with dominant RYR1 mutations. The underlying mutations and the heterogeneity in clinical symptoms, characterized primarily by the varying severity and extent of muscle weakness, suggest a complex underlying pathophysiology (4,5). In spite of the broad clinical features of RyR1-related myopathies, the pathology is dominated by ultrastructural derangement of myofibres resulting in the focal loss of mitochondria in either a single longitudinal central core or multiple smaller cores (multi-minicores) (6). Accordingly, a central question of the pathophysiology of RyR1-related myopathies is the identification of pathways linking RyR1 mutations to mitochondrial dysfunction and loss.

Given the central role of RyR1 in Ca^2^^+^-mediated excitation-contraction (EC) coupling in skeletal muscle, significant research has focussed on the consequences of mutations on this process. The emerging picture divides the functional outcome into (i) defects in mechanical coupling in the triad structure and increased Ca^2+^ leak through RyR1 channels, mostly characterizing dominant mutations, and (ii) loss of RyR1 function, often associated with recessive mutations leading to reduced expression of RyR1 (7,8). Mapping of mutations on the complex structure of the RyR1 channel also revealed a characteristic pattern. Dominant (gain-of-function) mutations tend to affect three domains: 1 (N-terminal), 2 (central) and 3 (C-terminal), and associated with CCD and/or the MHS and exertional rhabdomyolysis traits. On the other hand, recessive mutations are distributed along the whole length of the RyR1 sequence, leading to loss of function, reduced expression or truncated proteins, clinically often resulting in MmD (9). How these heterogeneous mutations lead to this range, development and interconversion of pathologies is not known (10–12). As a direct consequence of RyR1 mutations on muscle fibre Ca^2+^ signalling, a wide range of effects have been described. Increased Ca^2+^ leak can lead to reduced SR Ca^2+^ content or consequent increased cytoplasmic Ca^2+^ levels. On the other hand, reduced RyR1 function can also result in reduced cytoplasmic Ca^2+^ levels, making it difficult to find a common Ca^2+^ signal-related pathomechanism that results in the development of cores.

Crucially, core formation has been linked to mitochondrial dysfunction, and has been observed in both core myopathy patient samples and experimental models of the disease (13–16). However, whether mitochondrial dysfunction is primary to core development or the consequence of the molecular derangement of the subtle molecular architecture of muscle fibres still remains unanswered. The relationship between mitochondrial dysfunction and altered Ca^2+^ signalling in RyR1 mutant myofibres can be envisaged in different ways. On the one hand, it has been shown that increased IP3R expression occurs in myotubes and human cell lines with hypomorphic RyR1 mutations disrupting EC coupling (17,18). This might represent an adaptative rearrangement of Ca^2+^ signal-related gene expression patterns (19), altering cytoplasmic or nuclear signalling, similarly as described in cardiomyocytes, where IP3Rs play a privileged role to directly trigger nuclear Ca^2+^ signals (20). Thus either directly, or indirectly, altered RyR1 and IP3R expression can be part of feedback loops of an altered Ca^2^^+^-dependent transcriptional network, which has been shown to affect mitochondrial gene expression, biogenesis and function in muscle (21,22). On the other hand, increased IP3R expression might also lead to increased direct transfer of Ca^2+^ between the SR-localized IP3Rs and mitochondria (23). Localization of a mitochondrial population (24) and IP3R/F_1_F_o_-ATP synthase immunofluorescence to the ‘intercisternal’ (25) might favour this signalling, promoting direct mitochondrial Ca^2+^ transfer from the SR. While mitochondrial Ca^2+^ uptake contributes to metabolic signalling and adaptation through altering NAD(P)H redox state and increasing ATP production, long-term Ca^2+^ overload can lead to mitochondrial permeability transition (26–28), an established pathomechanism in muscle disease (29). Alternatively to IP3Rs, hyperactive RyR1 itself, even if almost exclusively localized to the triads, may also be associated with mitochondrial Ca^2+^ overload (30).

Here we have tested the hypothesis that IP3R-mediated mitochondrial Ca^2+^ signalling and accompanying alterations of mitochondrial gene expression patterns underlie the functional phenotype of core myopathies. In order to find potential associations between specific RyR1 mutations, gene expression patterns and signalling phenotypes, we have used patient biopsy material with a range of RyR1 mutations and mouse cellular and *in vivo* models with altered RyR1 expression and RyR1 mutations. Our findings reveal a complex relationship between loss of RyR1, increased IP3R-mediated cytoplasmic-nuclear and mitochondrial Ca^2+^ signalling, and altered mitochondrial gene expression and function and indicates an adaptive phenotype, presumably driven by altered Ca^2+^ signalling.

## Results

### Selected patient material

**Figure 1.**
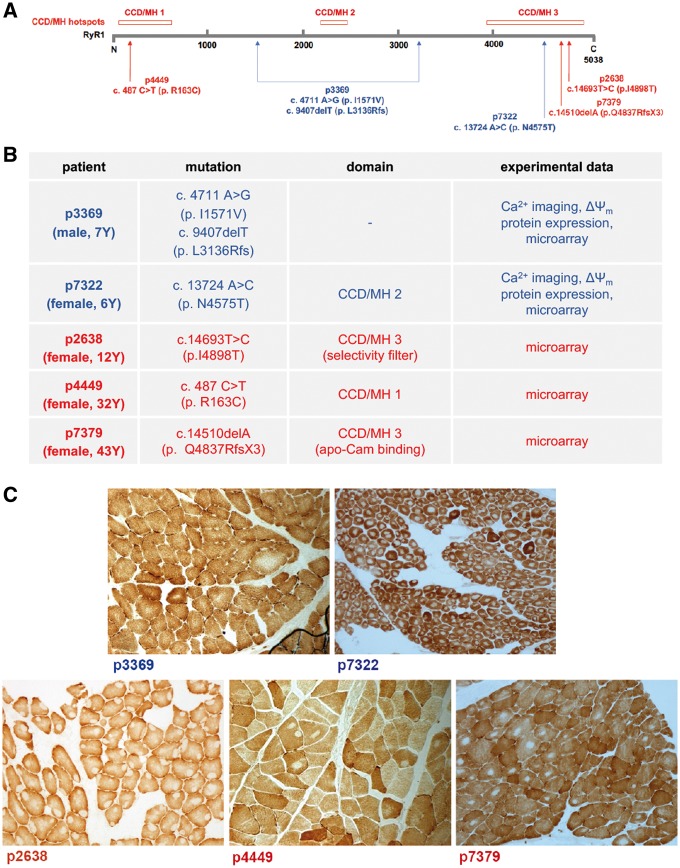
Patient material used in the study. Positions (**A**) and characteristics (**B**) of RyR1 mutations detected in patients with CCD and MmD. Muscle biopsies for microarray analysis were used from all patients, while primary myotubes were generated from two patient biopsies (shown in blue). (**C**) Representative histopathologic changes in specific *RYR1*-related myopathies included in the study. Wide-field light microscopy images of cytochrome *c* oxidase staining are shown. In p3369, absolute prevalence of type 1 fibres and areas of varying size devoid of oxidative enzyme stain; in the rest of the sections numerous cores of varying size and number are observed. In p7322 and p7379 cores are centrally, in p2638 peripherally and in p4449 both centrally and peripherally located.

In order to understand the consequences of RyR1 mutations in primary patient-derived tissues and differentiated myotubes, we have selected biopsies collected from patients with CCD and MmD with *RYR1* mutations established at the molecular level. For functional analysis on primary myotubes differentiated from patient-derived myoblasts, we have selected material from two affected children and controls matched for age and gender: (i) patient p7322 exhibiting CCD pathology due to a dominant mutation in the MH/CCD hotspot 3 (c.13724 A > C; p.N4575T) and (ii) patient p3369 with MmD pathology with compound heterozygous mutations in regions outside of MH/CCD hotspots presumably resulting in truncated non-functional RyR1 protein (c.A4711G/p.I1571V + c.9407delT/p.L3136Rfs). For gene expression studies, in addition to these prototypic MmD and CCD patients, a further affected child and two adult CCD patients were used with dominant mutations in MH/CCD hotspots 1 and 3 (p4449: c.487C > T/p.R163C, adult; p2638: c.14693T > C/p.I4898T, child; and p7379: c.14510delA/p.Q4837RfsX3, adult). The localization of the mutations and the summary of the patient material is shown in Figure 1, for clinical and pathological data, see Supplementary Material, Table S1.

### IP3-mediated cytoplasmic, nuclear and mitochondrial Ca^2+^ signalling is up-regulated in myotubes of patients with RyR1 mutations

**Figure 2.**
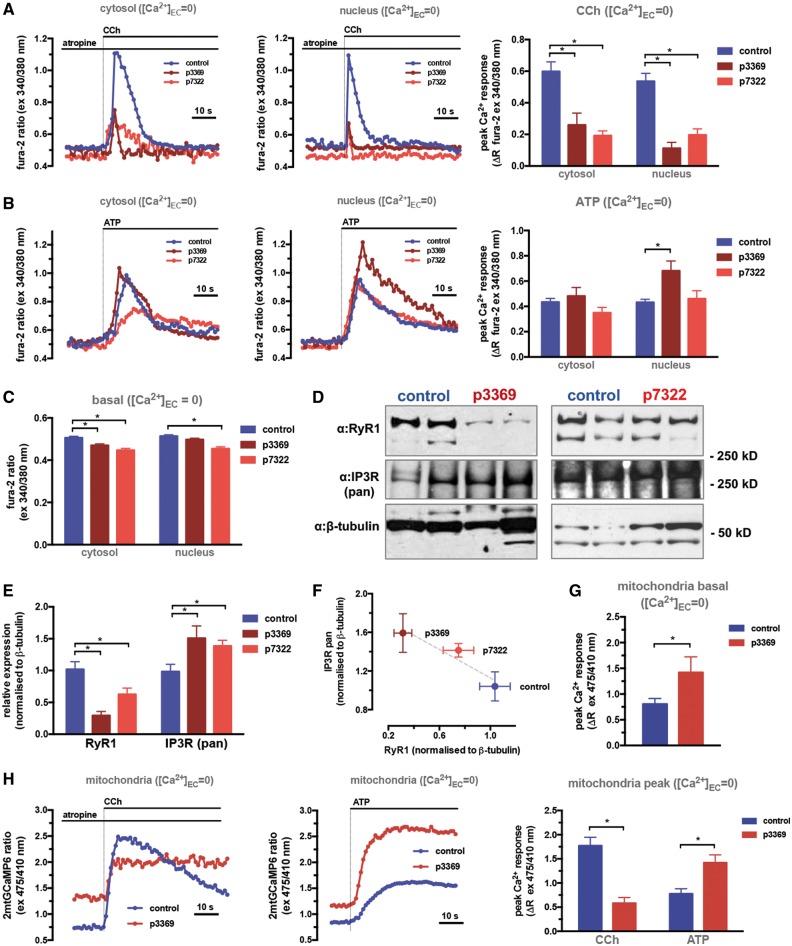
Up-regulation of IP3R-mediated Ca^2+^ signalling in CCD and MmD patient myotubes. Primary myotubes were generated from biopsies of CCD (p7322) and MmD (p3369) patients and age-matched controls (p7875, p6578, pooled data). Differentiated myotubes were loaded with the Ca^2+^-sensitive ratiometric dye fura-2 and fluorescent intensity ratios (ex: 340/380 nm, em: 505 nm) were measured using a wide-field fluorescent imaging system (see Materials and Methods section). (**A)** Response to CCh (0.5 µM) in the presence of atropine (10 µM) in Ca^2+^-free EC medium (supplemented with 200 µM EGTA) shows Ca^2+^ release mediated by RyR1. (**B)** Response to ATP (100 µM) in Ca^2+^-free EC medium (supplemented with 200 µM EGTA) shows Ca^2+^ release mediated by IP3Rs. Representative traces of ratios measured in cytosolic (left panels) and nuclear (middle panels) regions of interest, and statistics of quantified signals (peak—basal ratios, right panels, means ± S.E.M. of >20 cells from at least three separate experiments) are shown. Data were analysed using one-way ANOVA with Bonferoni *post-hoc* tests for multiple comparisons (*P < 0.05). (**C)** Quantification of basal fura-2 ratios, representing basal [Ca^2+^] in control and patient myotubes. (**D)** Western blot analysis of patient and control myotube protein extracts as described in the Materials and Methods section. Duplicates of 20 ug protein extracts are shown. (**E)** Quantification of signals from western blot analysis. Signals from the IP3R and RyR1 bands were normalized to β-tubulin, means ± S.E.M. of >3 replicates are shown. Data were analysed using two-way ANOVA with Bonferoni *post-hoc* tests for multiple comparisons (*P < 0.05). (**F)** Correlation plot of RyR1 and IP3R intensities from the same samples. (**G**, **H)** For analysis of mitochondrial Ca^2+^ signals, myotubes were infected with the adenoviral recombinant mitochondria-targeted Ca^2+^ probe 4mtGCaMP6 expression construct, and ratios (ex: 475/410, em: 520 nm) were imaged as described in the Materials and Methods section. **(G)** shows basal ratios in controls and the p3369 MmD patient. (**H)** Response to CCh (0.5 µM) in the presence of atropine (10 µM) in Ca^2+^-free EC medium (supplemented with 200 µM EGTA) shows Ca^2+^ release mediated by RyR1 (left panel) and response to ATP (100 µM) in shows Ca^2+^ release mediated by IP3Rs (middle panel). Quantified peak-basal values are shown in the right panel. Data were analysed using two-way ANOVA with Bonferoni *post-hoc* tests for multiple comparisons (*P < 0.05).

To characterize RyR1- and IP3R-mediated Ca^2+^ fluxes in patient cells carrying RyR1 mutations that underlie CCD and MmD pathologies, we first evaluated nuclear and cytosolic Ca^2+^ signals in primary differentiated myotubes obtained from muscle biopsies. Cells were loaded with the Ca^2^^+^-sensitive fluorescent ratiometric dye fura-2-AM. RyR1-mediated Ca^2+^ signals were induced by the nicotinic acetylcholine (ACh) receptor agonist carbachol (CCh, 0.5 μM) in the presence of the muscarinic ACh receptor blocker atropine (Atr, 10 μM) which activates the dihydropyridine receptor (DHPR, CaV1.1)-RyR1 axis. IP3R-mediated Ca^2+^ release was triggered by the G-protein coupled receptor agonist ATP (100 μM). Myotubes were stimulated in the absence of extracellular (EC) Ca^2+^ (medium supplemented with 200 μM EGTA) (Fig. 2A–C) or in the presence of EC Ca^2+^ (1 mM) (Supplementary Material, Fig. S1A and B) to discriminate between Ca^2+^ signals generated by SR Ca^2+^ release and by the sum of Ca^2+^ influx and SR release pathways, respectively. Since myotubes are not fully differentiated muscle fibres, Ca^2+^ influx induced by CCh contributes significantly to the response in the presence of EC Ca^2+^, similarly to the ATP-mediated response, which can be mediated not only by IP3-coupled P2Y but also by the ligand-activated P2X channels (19). Thus, in order to specifically address Ca^2+^ release from intracellular stores, we have primarily evaluated the contribution of RyR1 and IP3Rs in the absence of EC Ca^2+^ (Fig. 2).

In myotubes derived from patients with MmD and with CCD, we found significant down-regulation of CCh-induced SR Ca^2+^ release in the cytosol which was reflected by a similar down-regulation when Ca^2+^ signals were quantified over the nuclear regions of interest (ROIs) (Fig. 2A), indicating impaired RyR1 function. Basal cytosolic [Ca^2+^], measured by basal fura-2 ratio values, also showed a small, but significant decrease in cells from both patients (Fig. 2C). The reduction of RyR1-mediated Ca^2+^ release correlated with significant decrease in RyR1 expression, quantified by western blotting of protein extracts from patient derived myotubes (Fig. 2D). While RyR1-mediated Ca^2+^ signals were reduced in both the MmD and CCD patient myotubes, interestingly, IP3-mediated responses showed an opposite trend. While no significant alterations were observed in the overall cytosolic Ca^2+^ signals induced by ATP, the myotubes from the patient with MmD (p3369) showed significantly increased nuclear Ca^2+^ signals compared to controls (Fig. 2B). The myotubes from the patient with CCD (p7322) also showed elevated nuclear Ca^2+^ signals in the presence of EC Ca^2+^ (Supplementary Material, Fig. S1B). To address the origin of the reciprocal regulation of RyR1- and IP3R-mediated Ca^2+^ signals, we quantified the expression of IP3Rs by western blotting using a pan-IP3R antibody (Fig. 2D–F). Importantly, IP3R expression increased in negative correlation with reduction of RyR1 protein levels, showing the highest expression in the MmD (p3369) patient muscle biopsy tissue.

To test whether increased IP3R-mediated Ca^2+^ signalling results in increased mitochondrial Ca^2+^ uptake in myotubes from the patient with MmD (p3369), we transfected cells with 2mtGCaMP6m, a recombinant fluorescent Ca^2+^ probe specifically targeted to mitochondria (31). The high dynamic range of this ratiometric probe allows the measurement of both basal and stimulated Ca^2+^ levels in the mitochondrial compartment. As shown in Figure 2G, p3369 patient myotubes had significantly increased basal mitochondrial [Ca^2+^], indicating enhanced Ca^2+^ transfer into the organelle. Moreover, enhanced Ca^2+^ transfer was due to IP3R-mediated Ca^2+^ release, since stimulation of the p3369 patient myotubes with CCh resulted in significantly reduced mitochondrial Ca^2+^ transients as compared with controls, but ATP stimulation led to significantly increased mitochondrial Ca^2+^ uptake (Fig. 2H).

These results suggest that remodelling of Ca^2+^ signalling in patient-derived myotubes involves up-regulation of IP3Rs in response to loss of RyR1, which drives increased IP3-mediated signalling in both the nuclear compartment and mitochondria.

### RyR1 knock-down in C2C12 mouse myotubes mimics remodelling of Ca^2+^ signalling in patient myotubes and increases IP3-mediated mitochondrial Ca^2+^ uptake

**Figure 3.**
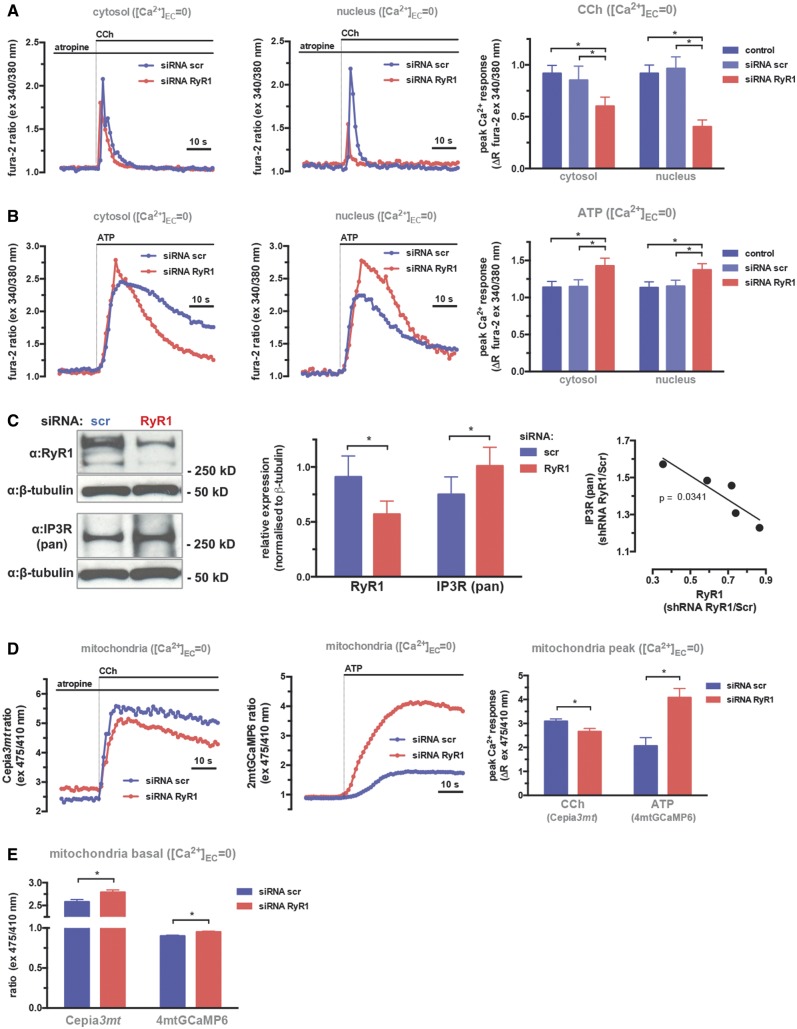
RyR1 silencing in C2C12 mouse myotubes stimulates IP3R-mediated Ca^2+^ signals in the cytosol, nucleus and mitochondria. Myotubes were differentiated from C2C12 cells transfected with scrambled (siRNA scr) or RyR1-specific (siRNA *RYR1*) siRNAs, or left untransfected. Differentiated myotubes were loaded with the Ca^2+^-sensitive ratiometric dye fura-2 and fluorescent intesity ratios (ex: 340/380 nm, em: 505 nm) were measured using a wide-field fluorescent imaging system (see Materials and Methods section). (**A)** Response to CCh (0.5 µM) in the presence of atropine (10 µM) in Ca^2+^-free EC medium (supplemented with 200 µM EGTA) shows Ca^2+^ release mediated by RyR1. (**B)** Response to ATP (100 µM) in Ca^2+^-free EC medium (supplemented with 200 µM EGTA) shows Ca^2+^ release mediated by IP3Rs. Representative traces of ratios measured in cytosolic (left panels) and nuclear (middle panels) regions of interest, and statistics of quantified signals (peak—basal ratios, right panels, means ± S.E.M. of >20 cells from at least three separate experiments, are shown. Data were analysed using one-way ANOVA with Bonferoni *post-hoc* tests for multiple comparisons (*P < 0.05). (**C)** Western blot analysis of protein extracts from siRNA scr and siRNA *RYR1-*transfected cells. About 20 ug protein extracts were analysed as described in the Materials and Methods section (left panel). Quantification of signals from western blot analysis is shown in the middle panel. Signals from the IP3R and RyR1 bands were normalized to β-tubulin, means ± S.E.M. of >3 replicates are shown. Data were analysed using two-way ANOVA with Bonferoni *post-hoc* tests for multiple comparisons (*P < 0.05). Correlation plot of RyR1 and IP3R intensities from the same samples is shown on the right panel. (**D)** For analysis of mitochondrial Ca^2+^ signals, C2C12 cells were co-transfected with siRNA scr or RyR1 and the recombinant mitochondria-targeted Ca^2+^ probes 4mtGCaMP6 or Cepia*3mt*, and ratios (excitation 475/410, emission 520 nm) were imaged as described in the Materials and Methods section. Response to CCh (0.5 µM) in the presence of atropine (10 µM) in Ca^2+^-free EC medium (supplemented with 200 µM EGTA) shows Ca^2+^ release mediated by RyR1 (left panel, Cepia*3mt* probe) and response to ATP (100 µM) in Ca^2+^-free EC medium (supplemented with 200 µM EGTA) shows Ca^2+^ release mediated by IP3Rs (middle panel, 4mtGCaMP6 probe). Statistics of quantified signals (peak—basal ratios, right panels, means ± S.E.M. of >20 cells from at least three separate experiments) are shown on the right panel. Data were analysed using one-way ANOVA with Bonferoni *post-hoc* tests for multiple comparisons (*P < 0.05). (**E)** Quantification of basal ratios of 4mtGCaMP6 or Cepia*3mt*, representing basal mitochondrial [Ca^2+^] in siRNA scr and *RYR1*-transfected myotubes. Data were analysed using one-way ANOVA with Bonferoni *post-hoc* tests for multiple comparisons (*P < 0.05).

Since we have observed loss of RyR1 in both MmD and CCD patient-derived myotubes, in order to directly address the role of RyR1 in remodelling myotube Ca^2+^ signalling, we used the C2C12 mouse myotube model where RyR1 levels were directly reduced using siRNA mediated silencing. Following RyR1 knock-down after transfection with *RYR1* siRNA, RyR1- and IP3R-mediated Ca^2+^ signals were measured using the protocols described above for patient myotubes. Results from myotubes stimulated in the absence of EC Ca^2+^ (medium supplemented with 200 μM EGTA) are shown in Figure 3A and B, while results in the presence of EC Ca^2+^ are shown in Supplementary Material, Figure S1C–G.

Altogether we found that in mouse C2C12 myotubes, the loss of RyR1 following its knockdown with siRNA *RYR1* led to significantly enhanced IP3-mediated Ca^2+^ release, in both the cytosolic and nuclear compartments (see Fig. 3A and B), confirming the role of RyR1 loss in the Ca^2+^ signalling pattern observed in patient myotubes. Moreover, quantification of RyR1 and IP3R protein expression levels by western blotting revealed similar inverse correlation between siRNA-mediated down-regulation of RyR1 and increased IP3R protein expression levels (Fig. 3C), pointing to a conserved mechanism of gene regulation patterns following RyR1 loss in mouse and human muscle and myotubes. Finally, in this model system, specific blockade of IP3Rs using Xestospongin-B (XeB) (32), confirmed that IP3Rs exclusively contribute to ATP but not to CCh-mediated Ca^2+^ signals (Supplementary Material, Fig. S1G).

Consequently, the similar behaviour of the C2C12 mouse and patient-derived human myotube models allowed us to use the mouse cell line to test the hypothesis that, following RyR1 loss, the increased IP3-mediated Ca^2+^ release is transferred to mitochondria. In order to measure mitochondrial Ca^2+^ in both low and high [Ca^2+^] ranges, we used two different recombinant fluorescent Ca^2+^ probes, specifically targeted to mitochondria, 2mtGCaMP6m (see Fig. 2G and H), and Cepia*3mt* (33), which were suitable to measure the range of ATP- and CCh-induced mitochondrial Ca^2+^ signals, respectively. Responses from myotubes stimulated in the absence of EC Ca^2+^ (medium supplemented with 200 μM EGTA) are shown in Figure 3D and E, while responses in the presence of EC Ca^2+^ are shown in Supplementary Material, Figure S1E and F. Overall, mitochondrial Ca^2+^ uptake followed the changes seen in nuclear and cytosolic Ca^2+^ signals. In *RYR1* siRNA-transfected mouse myotubes, ATP-induced, IP3R-mediated mitochondrial Ca^2+^ uptake was significantly increased compared with control cells, while the RyR1-mediated, CCh-induced signals were significantly reduced (Fig. 3D). Importantly, the IP3R-mediated mitochondrial Ca^2+^ uptake increased more than twofold, while RyR1-mediated mitochondrial Ca^2+^ signals were reduced only by ∼10%, indicating a stronger coupling of mitochondria to IP3Rs in this cellular model. The coupling to the increased levels of IP3Rs led also to an increase in basal mitochondrial [Ca^2+^], confirmed using both mitochondria-targeted recombinant Ca^2+^ probes (Fig. 3E). Altogether, these results demonstrated large-scale remodelling of global and compartmentalized cellular Ca^2+^ signalling following loss of RyR1, which thus can be a common phenotype linking recessive MmD and a subset of dominant mutations associated with CCD patients.

### Bioenergetic consequences of altered Ca^2+^ signalling in myotubes with loss of RyR1

**Figure 4.**
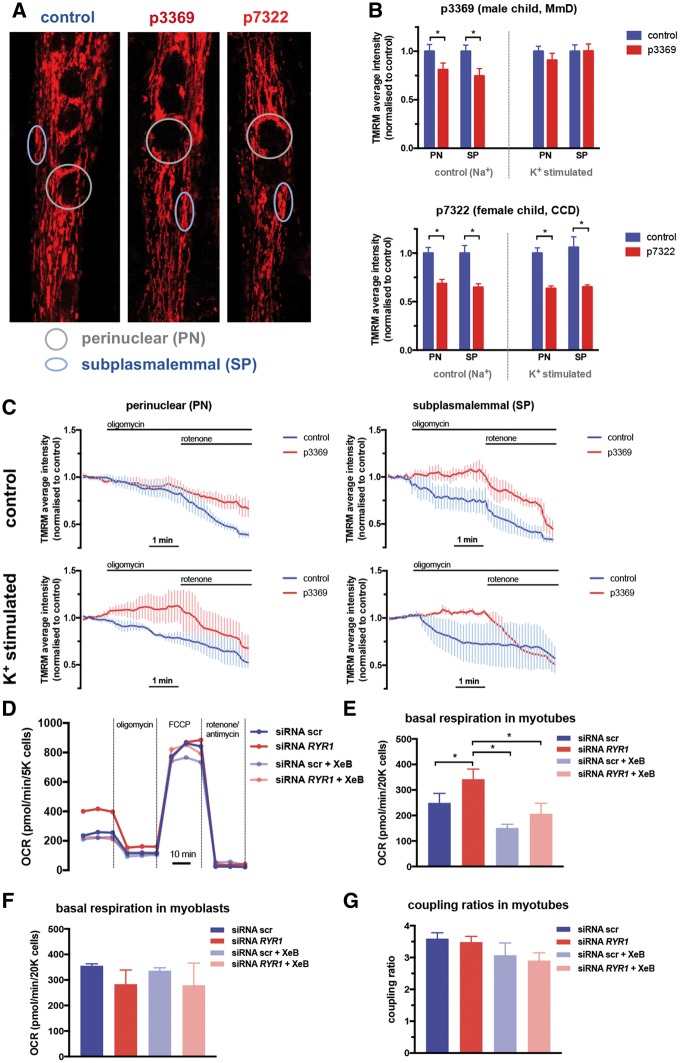
Mitochondrial bioenergetics in CCD and MmD patient derived and in C2C12 myotubes following *RYR1* silencing. Primary myotubes, generated from biopsies of CCD (p7322) and MmD (p3369) patients and age-matched controls (p7875, p6578, pooled data) were imaged in the presence of 4 nM TMRM and 0.8 µM cyclosporin-H, following 30 min incubation to reach steady-state distribution of the dye. Cells were pre-incubated and imaged in control ([Na^+^] = 140 mM) or K^+^ containing ([Na^+^] = 70 mM, [K^+^] = 70 mM) depolarizing recording medium, using a confocal system (ex: 535 nm, em: >560 nm) as described in the Materials and Methods section. (**A)** Representative images of control and patient cells, showing examples of perinuclear (PN) and SP regions of interests where average TMRM intensities were quantified. (**B)** Steady-state TMRM intensities in p3369 MmD (upper panel) and p7322 CCD (lower panel) patient myotubes. Means ± S.E.M. of >20 cells from at least three separate experiments are shown. (**C)** Sensitivity of mitochondrial membrane potential to inhibition of the F_1_F_o_ ATP synthase with oligomycin (10 µM) and complex I of the respiratory chain with rotenone (2 µM) was measured in time-lapse imaging of TMRM-loaded cells as in (A). Values from PN and SP regions of interests were normalized to the average of the initial 5 time points. Means ± S.E.M. of >20 cells from at least three separate experiments are shown under control (Na^+^, upper panels) and K^+^ stimulated (lower panels) conditions. Data were analysed using one-way ANOVA with Bonferoni *post-hoc* tests for multiple comparisons (*P < 0.05). (**D–G)** OCR was measured in C2C12 mouse myoblasts (F) and differentiated myotubes (D, E, G) in a Seahorse XF-24 EC flux analyser. Cells were transfected with siRNA scr or *RYR1*, and treated with XeB (2 μM) 30 min prior to measurements. (D) Basal OCR (pMoles/min/20 K seeded cells) were recorded followed by injection of 2 μM oligomycin to measure coupled respiration, 0.8 μM FCCP to induce maximal oxygen consumption. Rotenone and antimycin A (1 μM each) were injected to assess non-mitochondrial OCR at the end of the experiment. Data are normalized to these values in each run. (E) Quantification of basal respiration. (F) As controls, non-differentiated myoblasts were treated and measured in the same way. Values were corrected to non-mitochondrial respiration, i.e. by subtracting OCR values after rotenone/antimycin-A addition. (G) Coupling ratios of respiration in myotubes, calculated as the ratio of basal and oligomycin-treated OCR. Data were analysed using one-way ANOVA with Bonferoni *post-hoc* tests for multiple comparisons (*P < 0.05).

In order to understand the functional consequences of the remodelled compartmental Ca^2+^ signalling in RyR1-deficient human and mouse models, we evaluated mitochondrial function and gene expression. First, since material available from patient biopsies was insufficient to carry out measurements of oxygen consumption measurements, we used live cell confocal imaging to measure mitochondrial membrane potential (Δ*ψ*_m_) in the CCD and MmD patient-derived myotube models. For these measurements, we used the membrane permeable cationic dye tetramethyl rhodamine methyl-ester at low concentration (TMRM, 5 nM), and quantified average intensities in two distinct subcellular mitochondrial populations, in the perinuclear and sub-plasmalemmal (SP) regions, since in differentiated myofibres the proteomic composition and function of SP mitochondria differs from the interfibrillar population (24). In addition to quantifying steady-state TMRM intensities, which reflect steady state Δ*ψ*_m_, to evaluate the contribution of the electron transport chain and the forward and reverse mode of the F_1_F_o_-ATPase in generating and maintaining Δ*ψ*_m_, we performed time-lapse measurement of single cells following the addition of oligomycin and rotenone to inhibit the F_1_F_o_-ATPase and respiratory complex I, respectively. In addition, in order to evaluate the effect of Ca^2+^ load on Δ*ψ*_m_, we have performed these experiments in both resting conditions and following stimulation of myotubes with elevated EC [K^+^], which by depolarizing the plasmamembrane, activates the EC coupling mechanism to release SR Ca^2+^ via RyR1 receptors. Figure 4A shows representative images of TMRM-loaded control and CCD/MmD patient-derived myotubes. Measurement of average TMRM intensities showed reduced steady-state Δ*ψ*_m_ in both patient groups, which difference, however, was almost completely abolished in the MmD patient (p3369) following K^+^ stimulation (Fig. 4B). Lower steady-state Δ*ψ*_m_ might indicate decreased respiratory chain activity or increased dissipation of the H^+^ gradient through the F_1_F_o_-ATPase or uncoupling related to mitochondrial damage. To differentiate between these possibilities we used the above described time-lapse protocol (Fig. 4C). Interestingly, unstimulated control myotubes showed slow loss of potential following inhibition of the F_1_F_o_-ATPase by oligomycin, indicating that glycolytic ATP was used to maintain Δ*ψ*_m_ in these cells. However, oligomycin treatment caused a small but significant hyperpolarization of the mitochondria in patient cells, indicating an intact respiratory chain and use of the F_1_F_o_-ATPase in the forward mode, producing mitochondrial ATP. These differences between control and patient myotubes were more pronounced in the SP population of mitochondria, but became clear following K^+^ stimulation also in the perinuclear region. Similar results were obtained in the CCD (p7322) patient myotubes (data not shown). Altogether these data indicate that the rate of mitochondrial ATP synthesis is higher and make a greater contribution to cellular ATP demand in patient myotubes. Importantly, no differences between the recessive MmD and dominant CCD patient-derived myotubes were observed, suggesting that a shared feature, such as the loss of RyR1 is the underlying cause.

To verify that increased oxidative phosphorylation following RyR1 loss is a result of IP3R-mediated stimulation of respiratory chain activity, we measured oxygen consumption rate (OCR) in C2C12 mouse myotubes using the Seahorse technology. We have compared OCR in control and *RyR1* siRNA-transfected myotubes in the absence or presence of XeB, a specific inhibitor of IP3R-mediated Ca^2+^ release. As shown in Figure 4D and E, silencing of *RyR1* led to significantly increased basal respiration, which was prevented by treating cells with XeB. Increased respiration was specific differentiated myotubes (Fig. 4F), and was not due to uncoupling (Fig. 4G). No significant changes in ECAR were observed. These data demonstrated that the up-regulation of IP3R-mediated Ca^2+^ release following the loss of RyR1 protein results in enhanced mitochondrial bioenergetics.

### Consequences of altered Ca^2+^ signalling on mitochondrial and Ca^2+^ homeostasis-related gene expression

**Figure 5.**
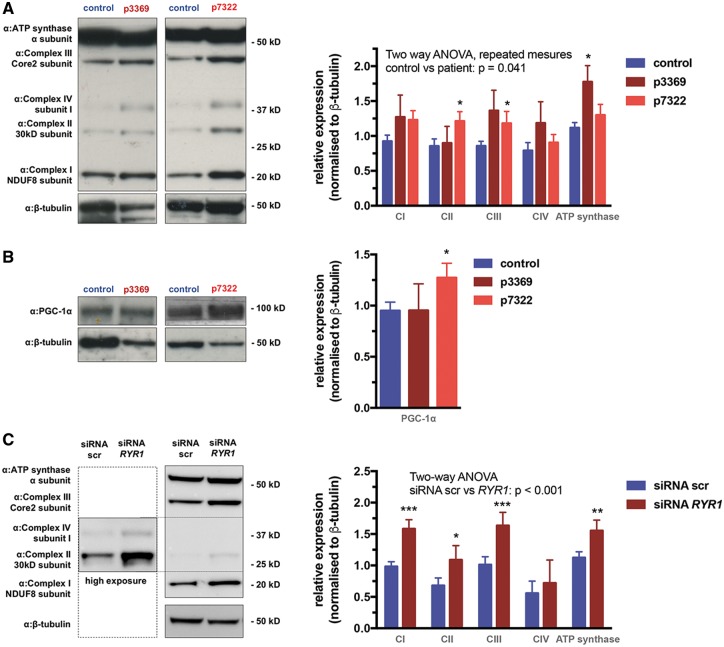
Increased mitochondrial biogenesis in CCD and MmD patient myotubes. Western blot analysis of subunits of respiratory chain complexes I–IV and the F_1_F_o_ ATP synthase (**A**) and PGC-1ɑ (**B**) from differentiated human patient myotubes or C2C12 mouse myotubes following transfection with siRNA scr or siRNA *RYR1* (**C**). About 20 ug protein extracts from differentiated myotubes were immunoblotted as described in the Materials and Methods section (left panel). Quantification of signals from western blot analysis are shown on the right panels. Signals from the respective bands were normalized to β-tubulin, means ± S.E.M. of >3 replicates are shown. Data were analysed using two-way ANOVA with Bonferoni *post-hoc* test. Asterisks denote significane between control and patient (A, B) or siRNA scr and siRNA *RYR1-*transfected cells (*P < 0.05, **P < 0.01, ***P < 0.001).

Increased mitochondrial oxidative function can be associated with enhanced IP3R-mediated Ca^2+^ signalling in at least two different ways. Direct IP3R-mediated Ca^2+^ transfer to mitochondria has been previously shown to contribute to maintenance and activation of the TCA cycle, respiratory activity and ATP production under both basal and stress conditions (34,35). Given the results shown in the sections on Ca^2+^ signalling (see Figs 2 and 3), this mechanism certainly plays an important role in the bioenergetic changes observed here. However, in both patient and mouse myotubes we found specific up-regulation of nuclear Ca^2+^ signals, which suggests that Ca^2^^+^-mediated changes in gene expression profiles might also take place. To address this hypothesis, we have applied two different approaches. First, we used immunoblots to assess the expression of respiratory chain components using the MitoProfile antibody set detecting single subunits of each complex, as well as PGC-1α, the best known nuclear co-regulator controlling mitochondrial biogenesis by co-activating a large set of nuclear-encoded mitochondrial genes (36,37). Importantly, we found significant up-regulation of respiratory complex subunits in the CCD (p7322) patient myotubes (complexes II–III), along with significant increase in PGC-1α content, and we have observed a similar trend in the MmD (p3369) patient cells, where the F_1_F_o_-ATPase α subunit was strongly induced (Fig. 5A and B). These results indicated activation of mitochondrial biogenesis in both patient myotubes. Similarly, respiratory subunits were significantly up-regulated in C2C12 mouse myotubes following silencing of *RyR1* expression (Fig. 5C), indicating that loss of RyR1 results in increased mitochondrial biogenesis, underlying improved oxidative phosphorylation (see Fig. 4).

**Figure 6.**
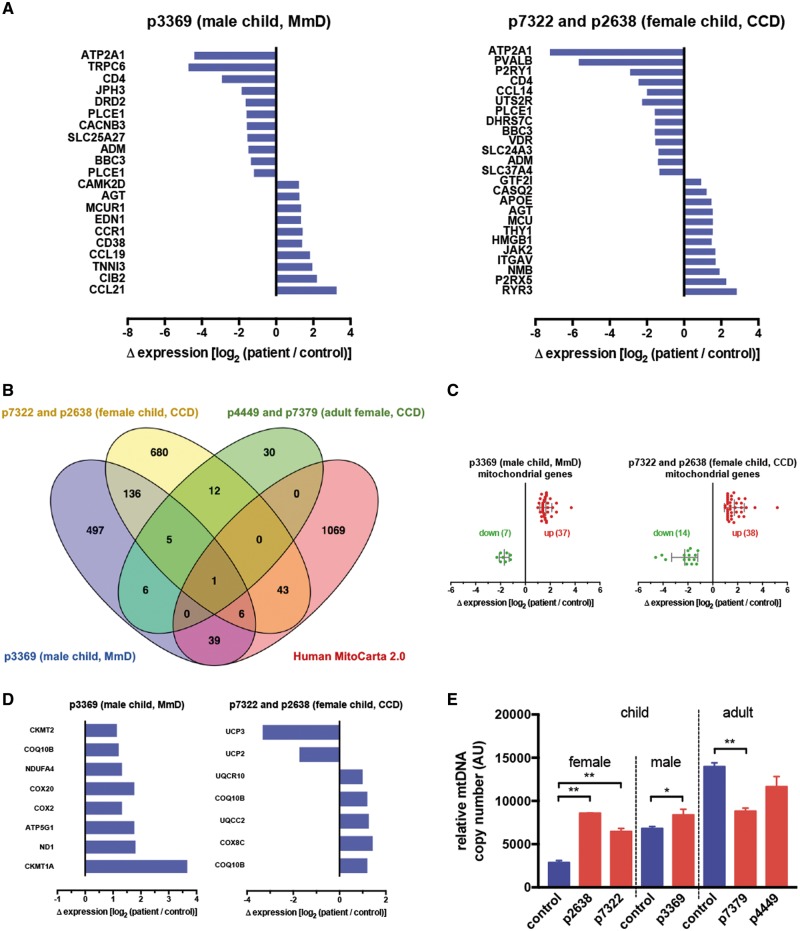
Transcriptome analysis of patient biopsies reveal remodelling of gene expression in Ca^2+^ signalling pathways and mitochondria. Total RNA extracts from male child (MmD, p3369), female child (CCDs, p7322 and p2638) and adult female (CCDs, p4449 and p7379) patient biopsies and age and sex-matched controls (p7875, p6578 and p8463, respectively), were used for microarray analysis as described in the Materials and Methods section. (**A)** Differentially expressed genes related to Ca^2+^ signalling pathways (GO: 0006874) identified by gene ontology analysis. Log 2 values of gene expression differences between p3369 MmD (left panel) and p7322 CCD (right panel) and respective controls are plotted. Consensus gene names are listed at the left. (**B)** Genes with significantly altered expression are shown in Venn diagrams in the human dataset. Overlaps between patients and with mitochondrial genes listed in the MitoCarta 2.0 database are shown. All differentially expressed genes and the Venn diagram intersections are listed in Supplementary Material, Data Set S1. (**C)** Differential expression values of mitochondrial genes in human patients. Log 2 values of up-regulated genes are shown in red, down-regulated genes are in green. (**D)** Differentially expressed mitochondrial genes coding for proteins of the respiratory chain and energy metabolism in human patient transcriptomes. Log 2 values of gene expression differences between p3369 MmD (left panel) and p7322 CCD (right panel) and respective controls are plotted. (**E)** Relative mtDNA copy number data from biopsies of all patients and age/sex-matched controls. Means  ± S.E.M. of three replicates are shown. Data were analysed using two-way ANOVA with Bonferoni *post-hoc* tests for multiple comparisons (*P < 0.05).

In order to verify and expand these findings in muscle tissue of human patient biopsies and in a mouse model of CCD/MmD, we performed microarray analysis. To represent patients at different ages and gender, we separately compared the transcriptomes of male child (MmD, p3369), female child (CCDs, p7322 and p2638) and adult female (CCDs, p4449 and p7379) patient biopsies to age- and sex-matched controls (p7875, p6578 and p8463, respectively). First, we identified differentially expressed gene sets (see Materials and Methods and Supplementary Material sections), which were further analysed for enrichments of specific cellular compartments and biological function (by gene ontology analysis using gProfiler (38) and for the presence of transcripts representing genes of Ca^2+^ signalling (http://www.ebi.ac.uk/QuickGO/term/GO: 0006874; date last accessed May 2, 2018) or mitochondrial pathways [MitoCarta 2.0 (39)]. In the male child MmD (p3369) patient, we found 690 transcripts which were differentially regulated (277 up, 413 down), while in the CCD female child (p7322 and p2638) patients 883 transcripts (504 up, 379 down) and in the CCD female adult (p4449 and p7379) patients 54 transcripts (23 up, 31 down) were significantly altered, indicating a large-scale remodelling of gene expression, in particular at a young age (see the full list of the differentially regulated genes in Supplementary Material, Data Set S1). Gene ontology analysis identified metabolism (in particular amino acid metabolism and oxidoreductase activity), oxygen transport and regulation of programmed cell death as key up-regulated biological processes, while glycolysis and myofibril assembly pathways were down-regulated. Importantly, significant enrichment of genes in the mitochondrial, SR and cytosolic compartments were equally present. Interestingly, remarkably less variations in gene expression and associated significant gene ontology terms were found in adult patient samples. Details of the gene ontology analysis are shown in Supplementary Material, Data Set S2. No alterations in IP3R and RYR1 gene transcripts were found in the patients indicating that RyR1 loss and the parallel increase in IP3Rs is the result of post-transcriptional mechanisms, most likely alterations of protein stability and degradation (40). However, highly significant alterations of other Ca^2+^ signaling-related genes were found (Fig. 6A), including almost complete down-regulation of ATP2A1 (SR Ca^2+^-ATPase 1, SERCA1), the SERCA isoform specific for fast fibres (21), most likely reflecting type 1 (slow) fibre predominance (11). Importantly, components of the mitochondrial Ca^2+^ uniporter (MCU and MCUR1), mediating Ca^2+^ uptake in the organelle, were up-regulated in both MmD (p3369) and CCD (p7322 and p2638) patients. Induction of mitochondrial Ca^2+^ transport proteins was accompanied by an overall increase in nuclear-encoded mitochondrial genes, as shown by the analysis of significantly altered genes intersecting with the human MitoCarta 2.0 list (Fig. 6B). The majority of these genes showed more than twofold increase in transcript abundance (Fig. 6C), and were enriched in genes coding for components of the respiratory chain in both the MmD and CCD child patient cohorts (p3369, p7322 and p2638, Fig. 6D). To test whether the elevated mitochondrial protein expression levels reflect increased mitochondrial content, we have assessed the relative mitochondrial DNA (mtDNA) content in patient biopsies. As shown in Figure 6E, both the MmD and CCD child patient cohorts (p3369, p7322 and p2638) also showed elevated mtDNA copy number, confirming that mitochondrial biogenesis I increased in the early stages of the disease.

In order to evaluate the hypothesis that gene expression changes are induced by RyR1 mutations, we performed microarray analysis in an established mouse model of RYR1-linked core myopathies, carrying the I4859T mutation in the RyR1 gene (16). We used muscle tissue extracts from two types of muscles of the heterozygous RyR^I4859T/wt^ mice with high and low mitochondrial content (*m. soleus*, type 1 and *m. extensor digitorum longus*, EDL, type 2A/2X, respectively). Similarly to the human core myopathy patient biopsies, we found alterations in a large gene set in both muscle types (soleus: 1039 transcripts differentially regulated, 579 up, 460 down; EDL: 974 transcripts differentially regulated, 502 up, 471 down, Supplementary Material, Data Set S1), which had a marked intersection with mouse MitoCarta gene set (Supplementary Material, Fig. S2A). Moreover, these nuclear-encoded mitochondrial gene transcripts also showed overall up-regulation with enrichment of respiratory chain components (Supplementary Material, Fig. S1B and C).

Altogether these results show that RyR1 dysfunction, silencing or loss, apart from direct consequences of RyR1-mediated Ca^2+^ signalling, leads to large-scale rearrangement of muscle gene expression, which involves both Ca^2+^ signalling and mitochondrial biogenesis pathways in human and murine samples. Further analysis of promoters of de-regulated genes also indicated a set of key transcription factors (TFs) responsible for the altered gene expression profile in human patients and animal models of RyR1-related core myopathies (see Supplementary Material, Data Set S3).

## Discussion

The RyR1 mutations analysed in this study were associated with loss of expression and function of the SR Ca^2+^ release channel. We have shown that loss of RyR1 leads to significant remodelling of skeletal muscle Ca^2+^ signalling, driven by up-regulation of the IP3R at post-translational level and a set of Ca^2+^ homeostasis-related proteins at the transcriptional level in both human and mouse myotubes. Dynamic regulation of IP3R levels by ubiquitination and degradation by the ER-associated degradation (ERAD) system has been recently established (40), and down-regulation of this pathways might provide explanation for increased IP3R levels following loss of RyR1 function. Indeed, we have observed significant down-regulation of several ubiquitin E3 ligases in human muscle samples and the overall ubiquitin-dependent protein catabolic process term (GO: 0006511) in the RyR^I4859T/wt^ mouse model (Supplementary Material, Data Set S3).

Previously, one study by Zhou *et al.* (17) has shown up-regulation of IP3Rs both at the transcriptional and proteins levels in patient-derived myotubes with recessive RyR1 mutations. Here, using microarrays from muscle biopsies we could not detect alterations of any of the ITPR1–3 genes, probably due to the low sensitivity of the method, compared with the quantitative PCR approach used in the previous study. Alternatively, up-regulation of IP3Rs might be present only in *in vitro* differentiated myotubes, which might represent an early stage of muscle fibre development. Importantly however, from functional point of view, here we demonstrated the first time that increased IP3R levels lead to elevated IP3-mediated Ca^2+^ signalling in both human and mouse myotubes, which affects the mitochondrial and nuclear compartments.

Although the general trend in all the models studied was up-regulation of IP3R-mediated Ca^2+^ signalling, the compartment specificity varied between different mutations and the C2C12 mouse myoblast models (see Figs 2 and 3) Moreover, when we measured Ca^2+^ signals in the presence of EC Ca^2+^, i.e. when either ATP- or CCh-induced Ca^2+^ influx was allowed in the system, the overall picture was more complex. In the patient cells, we observed significant down-regulation of CCh-induced Ca^2+^ signals in the cytosol of both patients and significant reduction of nuclear Ca^2+^ signals in myotubes from patient p3369 but not from patient p7322. On the other hand, we noted a significant up-regulation of ATP-induced nuclear Ca^2+^ signals in patient p7322, an effect which was missing in patient p3369, while cytosolic Ca^2+^ was not affected in any patients (see Supplementary Material, Fig. S1). However, a more homogenous up-regulation in all compartments was observed in C2C12 myotubes following *RYR1* silencing. These differences in the remodelling of Ca^2+^ signals might reflect different levels of the loss of RyR1 protein and function, as well as the different distribution of IP3R expression in the various cellular models. Whether remodelling of Ca^2+^ signalling pathways in fully differentiated myofibres have similar pattern, remains to be determined.

Apart from demonstrating the functional role of IP3Rs in remodelling compartmentalized Ca^2+^ signalling and increased mitochondrial Ca^2+^ uptake, our study provides the first evidence for altered mitochondrial gene expression and function as the consequence of RyR1 mutations associated with loss of the Ca^2+^ release channel. Mitochondrial damage due to dominant RyR1 mutations (41), disruption of mitochondrial distribution (13) has been previously shown in mouse models, zebrafish (15) and well documented in human muscle biopsies (6,5,11,42), but whether these alterations are the primary cause of core development and muscle pathology is not known. While direct damage of mitochondria due to excess Ca^2+^ leak has been previously proposed (30), our results indicate a different role for IP3R-mediated Ca^2+^ signals. Up-regulation of IP3R and concomitant increase in mitochondrial Ca^2+^ load appear to increase the metabolic competence of mitochondria in the myotube models of both CCD and MmD patients. Of note, muscle biopsies also showed up-regulation of members of the mitochondrial Ca^2+^ uptake machinery (MCU, MCUR1), indicating that increased mitochondrial Ca^2+^ uptake might also happen *in vivo*, as part of the pathological process in core myopathies. However, whether the localization of IP3Rs (25) and the (43) with respect to mitochondria in fully differentiated muscle fibres would allow this direct activation of mitochondrial metabolism remains to be shown. The direct, Ca^2^^+^-mediated activation of mitochondria in our models is also accompanied by increased mitochondrial biogenesis. Although no apparent adaptive proliferation of mitochondria has been previously described in CCD and MmD, selective up-regulation of genes involved in the respiratory chain and energy production by activation of Ca^2^^+^-dependent TFs or epigenetic changes (8) might provide adaptation to the overall loss of mitochondria in the core areas.

Our patient material comprised autosomal dominant cases, such as p4449/p7379, cases with dominant *de novo* mutations (p7322/p2638) and the autosomal recessive case p3369. The latter resulted in MmD while all other cases in CCD. Importantly, in both functionally studied cases (p3369/p7322) mutations led to loss of RyR1, which thus can accompany both AD- and AR-inherited disease, and both MMD and CCD histology. Moreover, since our study was limited to cases where RyR1 loss of protein and function dominated the pathomechanism, and thus partial silencing of the mouse RyR1 in C2C12 myotubes provided an appropriate model to study compartmentalized Ca^2+^ signalling. Interestingly, results from microarray studies of the AD patients showed partial overlap with the AR case, indicating that the observed mitochondrial phenotype is independent of the mode of inheritance. However, the consequences of mutations on RyR1 expression and function vary wildly in specific cases (10,17), thus whether IP3R-mediated Ca^2+^ signals operate as an overall adaptive mechanism in core myopathies remains to be studied. However, our study corroborated previous results showing negative correlation between the two main SR Ca^2+^ release channels, RyR1 and the IP3R. Moreover, by rendering an overall adaptive function most likely for the up-regulation of IP3Rs, the hypothesis of IP3-mediated Ca^2+^ overload as a ruling pathomechanism in RyR1-related myopathies is implausible.

## Materials and Methods

### Cell culture and transfection

Primary cultures of myoblasts from human quadriceps biopsies were obtained after collagenase A (Roche) digestion in 1% gelatin pre-coated dishes. Human myoblasts were grown in HAMF12 w/o glutamine medium (Euroclone), supplemented with 20% heat inactivated fetal bovine serum (FBS, Invitrogen), 100 µg/ml penicillin and streptomycin, 25 ng/ml fibroblast growth factor 2 (FGF2) (lowered to 5 nM for cell maintenance after second cell passage). Cells were incubated at 37°C under 5% CO_2_ and passaged 1: 5 or 1: 10 at 70% confluence. Myoblasts were differentiated into myotubes at 80% confluence in a medium containing Dulbecco’s modified Eagle medium (DMEM, Invitrogen) supplemented with 2% horse serum (HS, Invitrogen), 100 µg/ml penicillin and streptomycin, 30 µg/ml insulin; myoblasts differentiate into mature myotubes after 5 days in differentiation medium.

C2C12 mouse myoblasts were grown in DMEM (Invitrogen), supplemented with 10% heat inactivated FBS (Invitrogen), 100 µg/ml penicillin and streptomycin. Cells were cultured and myoblasts were differentiated using the same protocol as for human cells. Transfection of C2C12 myoblasts with siRNAs or with cDNAs was carried out with Lipofectamine 2000 (Invitrogen) according to the manufacturer’s instructions. After 6 h of transfection, the medium was replaced with DMEM supplemented with 10% FBS; 12 h later the medium was replaced with differentiation medium. Transfection efficiencies were tested by co-transfecting cells with GFP, and were typically > 80%. Myoblasts differentiate in mature myotubes after 5 days in differentiation medium. Cell passage numbers were all less than 15.

### Patient samples

Patient’s samples were selected by screening archived muscle biopsies hosted at the Neuromuscular Bank of Tissues and DNA samples of the University of Padova. Selection criteria were histopathological diagnosis of core myopathy, characterized *RYR1* gene mutations, the availability of clinical data and availability of cultured myoblast cell lines. Muscle biopsies were obtained at the time of diagnosis after signing of an informed consent. The study was carried out in accordance with the ethical rules and guidelines issued by the Ethical committee of the participating centres and with the declaration of Helsinki. Five biopsies were selected for the study.

### Mouse model

RyR1 I4895T knock-in mice on a 129S2/SvPasCrl background were kindly provided by the S. Hamilton laboratory, Texas, USA. These founder mice were rederived using C57BL/6J for breeding. This resulted in a new sub-line with a mixed genetic background (approximately 50% 129S2/SvPasCrl and 50% C57BL/6J after rederivation). Since homozygotes have a perinatal lethal phenotype (44), heterozygotes (HET) were crossed with wild-type (WT) littermates or C57BL/6J mice (Charles River) to maintain the colony. At least five such crosses had occurred before the experiments. Ear snips from adult mice and tail snips from sacrificed neonates were retained for genotyping. DNA was extracted from tissue samples using the Extract-N-Amp kit (Sigma) and PCR was used to amplify the fragment covering the mutation site. Forward and reverse primers were 5′-GGTCTTCCTGTCTCAATAACCCGATCTAGAAAC-3′ and 5′-GATGGAGAAACCAAAGCTCAGAGAGACCAC-3′, respectively. Since the inserted mutation site also contains an Age I target sequence, only samples containing the mutated allele undergo Age I digestion.

### Ca^2+^ imaging

Cells for imaging were seeded on 1% gelatin pre-coated 24 mm coverslips placed in six-well plates. Human-derived primary myoblasts were plated at a concentration of 2 × 10^6^ while C2C12 myoblasts were plated at a concentration of 1, 5 × 10^6^. To measure cytosolic and nuclear Ca^2+^ dynamics, differentiated cells were loaded with 2 µM Fura-2/AM (Life Technologies) for 20 min at 37° in modified Krebs-Ringer buffer (KRB) (135 mM NaCl, 5 mM KCl, 1 mM MgCl_2_, 20 mM HEPES, 1 mM MgSO_4_, 0.4 mM KH_2_PO_4_, 1 mM CaCl_2_, 5.5 mM glucose, pH 7.4). Images were acquired at 1/s (with exposure time set to 400 ms for all experiments) using alternate excitation at 380 and 340 nm and collecting emitted light with a 505/20 emission filter. To measure mitochondrial [Ca^2+^] in C2C12 myotubes we co-transfected myoblast with 3 µM of the mitochondrial targeted probes 2mtGCaMP6m or Cepia3mt and 1 µM siRNA-*RYR1* (with exposure time set to 200 ms for all experiments). Images acquisition rate was 1/s. Images were generated using a 475–415 excitation filter with exposure time set to 200 ms for 475 nm and 100 ms for 415 nm, emitted light was collected was collected through a 515/30 nm band-pass filter (Semrock). To measure mitochondrial Ca^2+^ levels in patient-derived differentiated myotubes we infected cells with an adenoviral 2mtGCaMP6m construct (1 ug/well in six-well plates) by adding the virus directly to the culture medium 36 h before performing the experiments. Imaging was performed using an Zeiss Axiovert 200 microscope equipped with a 63×/1.4 N.A. Plan Apochromat objective. Excitation was performed with a DeltaRAM V high-speed monochromator (Photon Technology International) equipped with a 75 W xenon arc lamp. Images were captured with a high-sensitivity Evolve 512 Delta EMCCD (Photometrics). The system was controlled by MetaMorph 7.5 (Molecular Devices) and was assembled by Crisel Instruments.

### [Ca^2+^] measurements with aequorin

C2C12 myoblasts were seeded on 1% gelatin pre-coated 13 mm coverslips placed in 24-well plates plated at a concentration of 4 × 10^4^. 4 days after addition of differentiation medium, cells were infected with 1 µM adenoviral cytosolic-targeted aequorin (cytAEQ). The following day (5th day in differentiation medium) coverslips with infected cells were incubated with 2 μM coelenterazine for 2 h in KRB supplemented with 1 mM CaCl_2_, and then transferred to the perfusion chamber. All luminometer measurements were carried out in KRB and agonists were added to the same medium. Experiments were terminated by lysing the cells with 100 μM digitonin in a hypotonic high [Ca^2+^] solution (10 mM CaCl_2_ in water), to discharge the remaining aequorin pool. The light signal was collected and calibrated into [Ca^2+^] values by an algorithm based on the Ca^2+^ response curve of aequorin at physiological conditions of pH, [Mg^2+^] and ionic strength (45).

### Western blot analysis

C2C12 siRNA *RYR1*-transfected and human-derived differentiated myotubes were harvested with ice-cold PBS and centrifuged 10 min at 4000 rpm at 4°C. Cellular extracts were prepared by solubilizing cells for 30 min in ice-cold lysis buffer (RIPA buffer 80%, 9% complete protease inhibitor cocktail tablets, Roche, 04693159001), 0.9% PMSF, 0.1% DTT, 9% phosphostop phosphatase inhibitor cocktail tablets, Roche 04906845001) supplemented with a cocktail of protease inhibitors (1%) (Sigma). Supernatants were collected after 25 min centrifugation at 12 000 rpm at 4°C. The total protein content was determined using the BCA protein assay (Thermo, 23209). A total of 20 µg proteins were loaded on a Nu-page 3–8% tris-acetate precast gel (Lifetechnologies) immersed in tris-acetate SDS running buffer (Lifetechnologies). Running was set up as follows: for IP3R and RyR1 30 min at 50 V and 2 h at 70 V; for mitochondrial respiratory complexes and PGC1-α 1 h 30 min at 130 V. Wet transfer was performed 2 h onto nitrocellulose (IP3R and RyR1) or methanol pre-activated PVDF membrane (Biorad) (mitochondrial respiratory complexes and PGC1-α). Membranes were blocked with 5% milk in TBS-Tween 1% for 1 h and incubated with primary antibodies overnight at 4°C in 1% milk in TBS-Tween 1%. We used the following antibodies: rabbit-IP3R (Santa Cruz, H-300, sc-28613), mouse RyR1 (Thermo, 34C, MA3–925), mouse mitoprofile (Mitosciences, MS604/H3941) rabbit PGC1-α (Santa Cruz, H-300, sc-13067), rabbit β-tubulin (Santa Cruz, H-235, sc-9104). Detection was carried out by incubation with secondary horseradish peroxidase-conjugated goat anti-rabbit or goat anti-mouse IgG antibodies (Biorad, 170–6515 and 170–6516, respectively) for 1 h at RT. Proteins were visualized by the chemiluminescent reagent ‘super signal west pico chemioluminescent substrate’ (Thermo, 34080). Densitometric analyses were performed by using ImageJ analysis software. We analysed by means of densitometric measurements of at least three independent experiments, normalized to β-tubulin.

### Mitochondrial membrane potential measurements

Human myoblasts were seeded at a density of 1.8 × 10^6^ on 1% gelatin pre-coated 24 mm coverslips. After 5 days in differentiation medium, mitochondrial membrane potential (Δ*ψ*_m_) was measured by confocal imaging. Cells were loaded with 4 nM tetramethyl-rhodamine-methyl-ester (TMRM, Life Technologies) for 30 min at 37°C in KRB (supplemented with 2 mM CaCl_2_) to measure Δ*ψ*_m_ at resting conditions (steady state) or loaded in a modified KRB with [NaCl] lowered to 70 mM and supplemented with 2 mM CaCl_2_ and 70 mM KCl. About 0.8 μM cyclosporine-H was also used in these experiments to reduce active TMRM extrusion. Z-stack images were acquired with a Leica Confocal SP5 microscope using a 100× immersion oil objective (Zeiss). TMRM was excited at 535 nm and emission was measured with a 560 nm LP filter. Eight bit images were taken every 20 s for 4 min with a fixed 1 s exposure time; laser power was set to 8%. Pinhole was set to 1.5 airy units and pixel size was set as 1024 × 1024 nm. ImageJ was used to quantify TMRM intensity on background-corrected images applying a constant threshold and quantifying the fluorescence intensity mean values in selected region of interests (see Fig. 5A).

### Oxygen consumption rate (OCR) measurements in C2C12 mouse myotubes and myoblasts

Low-passage C2C12 myoblast were seeded 20.000/well in a V7 XF 24-well cell culture microplate (XF24 EC flux assay kit, Seahorse, Bioscience) in culture medium. Cells were transfected (as reported in the previous section) with 0.1 ug/well siRNA RyR1 (Sigma) and 0.5 ul lipofectamine 2000 (Invitrogen) per well and the following day culture medium was replaced with differentiation medium. After 5 days in differentiation medium, mature myotubes were gently rinsed in measurement buffer (MB) consisting of DMEM (Sigma, D5030) 8.3 g/l, sodium pyruvate 1 mM (1.1 g/l), glucose 25 mM (4.5 g/l), NaCl 33 mM (1.85 g/l), phenol red (15 mg/l), l-Glut 10 ml/l, pH 7.4, and placed in a 37° humidified un-buffered incubator for 2 h to allow temperature and pH equilibration. Myotubes were visually inspected prior to and after MB addition then loaded onto the instrument. Bio-energetic analysis was performed utilizing an XF24–3 Seahorse Extracellular Flux Analyzer (Seahorse Bio-Science). After an equilibration step, basal OCR(pMoles/min) were recorded using 3 min mix, 2 min wait and 3 min measure (looped three times) cycles prior to the injection of 2 uM oligomycin to inhibit the ATP synthase. Three more measurement loops were recorded prior to injection of substrate plus 0.8 uM FCCP to induce maximal oxygen consumption. Following recording of three more measurement loops, rotenone 1 uM and 1 uM antimycin A (inhibitors of mitochondrial respiration) were injected to assess non-mitochondrial OCR. Stimuli prepared in MB (70 μl volumes) were pre-loaded and sequentially injected as indicated through ports in the XF24 calibration cartridge to final concentrations of 1 μg/ml oligomycin, 800 nM FCCP and 1 μM rotenone and antimycin-A. XeB (2 μM) was added to siRNA scr and siRNA RYR1-transfected cells 30 min prior to measurements, and was included in ports in the XF24 calibration cartridge to maintain final concentrations of 2 μM when injected. Since the number of differentiated cells in the culture was not feasible to determine, OCR values are expressed as pMoles/min/20 K seeded cells. As controls, non-differentiated myoblasts were treated and measured in the same way. Values were corrected to non-mitochondrial respiration, i.e. by subtracting OCR values after rotenone/antimycin-A addition.

### Quantification of mtDNA

Total DNA for mtDNA copy number estimation was extracted from muscle biopsies with Qiagen DNeasy Blood & Tissue kit according to the manufacturer’s protocol. The quantitative real-time PCR measurement of mtDNA copy number was conducted according to the protocol in Rooney *et al.* (46), by utilizing human sequence-specific primers targeting the mtDNA region coding for the tRNALeu (UUR) gene (F: CACCCAAGAACAGGGTTTGT; R: TGGCCATGGGTATGTTGTTA) and the nuclear region coding for the B2-microglobulin gene (F: TGCTGTCTCCATGTTTGATGTATCT; R: TCTCTGCTCCCCACCTCTAAGT). RT-PCR reaction parameters were set according to protocol in Rooney *et al.* (46), using SYBR© Green JumpstartTM TaqTM ReadyMixTM (Sigma) on a Biorad CFX96 Thermocycler. Ct analysis was conducted through the Biorad CFX manager software. For the determination of relative mtDNA copy number the following equations were used:

- ΔCt = Nuclear Ct – Mitochondrial Ct
- Relative mtDNA content = 2 × 2ΔCt

### Microarray analysis

Total mRNA was extracted from 40 mg of frozen human or mouse muscle tissues using 1 ml trizol per sample using a tissue lyser (Tissue lyser II, Qiagen). Gene expression studies were performed using Agilent technology with one-color (quick amp labelling) kit following the manufacturer’s instructions. Quantitative and qualitative analysis of total RNA were performed with NanoDrop spectrophotometers (Thermo Scientific) and Nanochip BioAnalyzer (Agilent). Human samples were analysed using the Human 4x44K V2 platform (Agilent) and data were normalized with the intra-array (Multiplicatively Detrended) and the inter-array (Quantile) methods. To evaluate the differences in transcript expression levels we used significance analysis of microarray (SAM) and we performed cluster analysis only on altered transcripts with false discovery rate (FDR) analysis of 0% using the complete linkage Euclidean method. EDL and soleus mouse samples were analysed using the Mus musculus 4x44K platform and data were normalized with the intra-array (Multiplicatively Detrended) and the inter-array (Quantile) methods. Differentially expressed transcripts were found using permutations of *t*-test with Bonferroni method (*P*-value  <  0.05). Enrichment analyses were performed using g: Profiler (38). To identify altered mitochondrial transcripts, we compared our results with The Human and Mouse MitoCarta 2.0 list (Broad Institute, Cambridge, MA, USA, https://www.broadinstitute.org/scientific-community/science/programs/metabolic-disease-program/publications/mitocarta/mitocarta-in-0) (39), using Venn diagrams (http://bioinfogp.cnb.csic.es/tools/venny/index.html). Microarray data have been deposited in GEO (GSE103855).

Putative TF-binding sites were determined using the TRANSFAC database (2015.3) (47) by a custom g: Profiler algorithm (http://biit.cs.ut.ee/gprofiler/help.cgi? help_id=21). Significant TFs were further analysed by listing all the genes ordered by how many TFs target them in the human patient and mouse model samples. In addition, we created heatmap plots of the genes ordered by this ranking and the TFs ordered by hierarchical clustering (see Supplementary Material, Data Set S3). In the heatmaps, blue signifies that the gene is regulated by the TF and white signifies that it is not. TFs belonging to the individual clusters were ranked according to significance values.

### Statistical analysis

Data are reported as mean ± S.E.M. Statistical differences were evaluated by ANOVA and *post-hoc* tests for multiple comparisons, or Student’s *t*-tests.

## Supplementary Material

Supplementary Material is available at *HMG* online.

## Supplementary Material

Supplementary Data
