# Supplementary material for: Inositol trisphosphate receptor-mediated Ca2+ signalling stimulates mitochondrial function and gene expression in core myopathy patients
Source: Hum Mol Genet. 2018 Apr 25;27(13):2367–82. doi: 10.1093/hmg/ddy149 (PMC6005141; doi:10.1093/hmg/ddy149)

Supplementary Data Set 2.a

p3369 (male child, MmD) upregulated genes

source	term name Gene Ontology (Biological process)	term ID	n. of term genes	n. of query genes	n. of common genes	corrected p-value
BP	single-organism metabolic process	GO:0044710	4320	224	72	3.33e-02
BP	small molecule metabolic process	GO:0044281	2128	221	48	3.44e-04
BP	organic acid metabolic process	GO:0006082	1123	179	28	3.87e-04
BP	oxoacid metabolic process	GO:0043436	1106	200	30	2.79e-04
BP	carboxylic acid metabolic process	GO:0019752	996	200	28	3.83e-04
BP	muscle contraction	GO:0006936	339	251	15	4.12e-02
BP	regulation of muscle contraction	GO:0006937	151	249	10	3.64e-02
BP	alpha-amino acid metabolic process	GO:1901605	219	197	11	1.54e-02

source	term name Gene Ontology (Cellular component)	term ID	n. of term genes	n. of query genes	n. of common genes	corrected p-value
CC	mitochondrion	GO:0005739	1710	255	44	3.16e-03
CC	mitochondrial part	GO:0044429	1007	255	30	1.28e-02

source	term name Gene Ontology (Molecular function)	term ID	n. of term genes	n. of query genes	n. of common genes	corrected p-value
MF	cofactor binding	GO:0048037	266	197	12	1.72e-02
MF	oxidoreductase activity	GO:0016491	756	217	22	2.53e-02

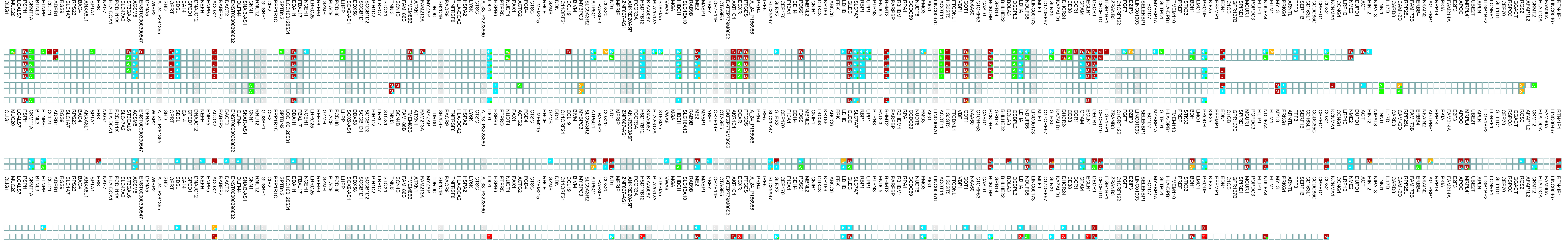

Supplementary Data Set 2.b

p3369 (male child, MmD) downregulated genes

source	term name<br>Gene Ontology (Biological process)	term ID	n. of term genes	n. of query genes	n. of common genes	corrected p-value
BP	response to wounding	GO:0009611	657	271	23	1.81e-02
BP	collagen metabolic process	GO:0032963	115	106	6	4.14e-02
BP	response to endogenous stimulus	GO:0009719	1453	209	32	1.00e-02
BP	cellular response to endogenous stimulus	GO:0071495	1249	209	29	1.11e-02
BP	response to growth factor	GO:0070848	658	225	25	3.11e-05
BP	response to fibroblast growth factor	GO:0071774	144	209	13	2.33e-06
BP	cellular response to growth factor stimulus	GO:0071363	632	212	21	1.53e-03
BP	cellular response to fibroblast growth factor stimulus	GO:0044344	137	209	10	1.60e-03
BP	regulation of signaling	GO:0023051	3136	367	82	3.41e-02
BP	response to reactive oxygen species	GO:0000302	217	361	16	1.61e-03
BP	response to hydrogen peroxide	GO:0042542	133	326	11	1.19e-02
BP	biological adhesion	GO:0022610	1360	210	37	7.45e-06
BP	cell adhesion	GO:0007155	1352	210	37	6.35e-06
BP	cell-substrate adhesion	GO:0031589	311	209	13	1.84e-02
BP	developmental process	GO:0032502	5966	367	143	4.66e-04
BP	anatomical structure development	GO:0048856	5547	367	139	4.45e-05
BP	tissue development	GO:0009888	1917	362	63	1.56e-04
BP	connective tissue development	GO:0061448	240	199	12	3.93e-03
BP	anatomical structure morphogenesis	GO:0009653	2449	367	85	4.46e-08
BP	anatomical structure formation involved in morphogenesis	GO:0048646	1009	367	43	3.96e-05
BP	regulation of developmental process	GO:0050793	2321	367	66	2.23e-02
BP	regulation of anatomical structure morphogenesis	GO:0022603	970	367	35	4.00e-02
BP	multicellular organismal process	GO:0032501	7202	367	167	1.39e-04
BP	system process	GO:0003008	2000	294	50	2.36e-02
BP	muscle system process	GO:0003012	428	223	21	5.10e-06
BP	muscle contraction	GO:0006936	339	77	11	5.27e-05
BP	striated muscle contraction	GO:0006941	167	77	7	4.43e-03
BP	regulation of multicellular organismal process	GO:0051239	2719	367	78	1.80e-03
BP	regulation of system process	GO:0044057	511	367	24	1.47e-02
BP	single-multicellular organism process	GO:0044707	6182	367	153	9.25e-06
BP	single-organism developmental process	GO:0044767	5877	367	140	1.07e-03
BP	muscle structure development	GO:0061061	609	362	26	2.45e-02
BP	multicellular organism development	GO:0007275	5102	367	132	1.80e-05
BP	regulation of multicellular organismal development	GO:2000026	1773	367	55	1.25e-02
BP	system development	GO:0048731	4543	367	122	1.08e-05
BP	skeletal system development	GO:0001501	488	209	16	3.47e-02
BP	animal organ development	GO:0048513	3322	209	56	6.93e-03
BP	muscle organ development	GO:0007517	372	362	19	3.60e-02
BP	circulatory system development	GO:0072359	975	367	43	1.45e-05
BP	cardiovascular system development	GO:0072358	649	367	31	4.15e-04
BP	vasculature development	GO:0001944	639	367	30	9.58e-04
BP	heart development	GO:0007507	519	361	23	4.31e-02
BP	animal organ morphogenesis	GO:0009887	999	362	38	3.33e-03
BP	blood vessel development	GO:0001568	613	367	30	3.95e-04
BP	blood vessel morphogenesis	GO:0048514	527	367	26	2.38e-03
BP	angiogenesis	GO:0001525	443	367	24	1.22e-03
BP	actin-myosin filament sliding	GO:0033275	39	77	4	2.52e-02
BP	muscle filament sliding	GO:0030049	39	77	4	2.52e-02
BP	regulation of cell death	GO:0010941	1536	365	50	8.11e-03
BP	regulation of programmed cell death	GO:0043067	1436	363	46	2.72e-02
BP	positive regulation of cell death	GO:0010942	628	365	28	5.78e-03
BP	regulation of apoptotic process	GO:0042981	1422	363	46	2.12e-02
BP	extracellular structure organization	GO:0043062	328	199	17	1.38e-05
BP	extracellular matrix organization	GO:0030198	327	199	17	1.31e-05
BP	response to oxygen levels	GO:0070482	366	365	20	9.00e-03
BP	response to decreased oxygen levels	GO:0036293	348	365	19	1.60e-02
BP	response to hypoxia	GO:0001666	340	365	18	4.27e-02
BP	intracellular signal transduction	GO:0035556	2621	365	73	1.07e-02
BP	signal transduction by protein phosphorylation	GO:0023014	900	361	35	5.14e-03
BP	MAPK cascade	GO:0000165	864	361	34	5.62e-03
BP	regulation of MAPK cascade	GO:0043408	668	361	30	1.62e-03
BP	regulation of cell communication	GO:0010646	3082	367	81	3.25e-02
BP	cell differentiation	GO:0030154	3880	367	99	8.32e-03
BP	movement of cell or subcellular component	GO:0006928	1922	367	57	3.15e-02
BP	cellular response to vitamin	GO:0071295	29	209	5	1.69e-02
BP	gas transport	GO:0015669	20	239	5	4.97e-03
BP	oxygen transport	GO:0015671	15	239	5	1.01e-03
BP	growth	GO:0040007	960	295	32	5.04e-03
BP	cellular response to chemical stimulus	GO:0070887	2757	228	52	1.44e-02
BP	response to mechanical stimulus	GO:0009612	209	287	13	1.04e-02
BP	regulation of cytokine biosynthetic process	GO:0042035	96	333	9	3.81e-02

source	term name<br>Gene Ontology (Cellular component)	term ID	n. of term genes	n. of query genes	n. of common genes	corrected p-value
CC	contractile fiber	GO:0043292	221	330	15	3.20e-03
CC	contractile fiber part	GO:0044449	205	330	15	1.23e-03
CC	myosin complex	GO:0016459	71	320	8	2.23e-02
CC	myosin filament	GO:0032982	22	32	3	1.11e-02
CC	myosin II complex	GO:0016460	34	320	6	2.20e-02
CC	muscle myosin complex	GO:0005859	27	320	6	5.27e-03
CC	hemoglobin complex	GO:0005833	12	239	4	1.60e-02
CC	endoplasmic reticulum lumen	GO:0005788	295	104	12	2.32e-05
CC	cytoplasm	GO:0005737	11137	368	225	1.18e-02
CC	extracellular matrix	GO:0031012	529	212	30	6.30e-12
CC	extracellular matrix component	GO:0044420	118	199	8	2.59e-02
CC	proteinaceous extracellular matrix	GO:0005578	363	212	26	2.22e-12
CC	collagen trimer	GO:0005581	98	103	6	1.35e-02

source	term name<br>Gene Ontology (Molecular function)	term ID	n. of term genes	n. of query genes	n. of common genes	corrected p-value
MF	sulfur compound binding	GO:1901681	219	209	13	3.61e-04
MF	glycosaminoglycan binding	GO:0005539	199	209	15	1.44e-06
MF	heparin binding	GO:0008201	150	288	15	3.46e-06
MF	extracellular matrix structural constituent	GO:0005201	79	212	7	2.54e-02
MF	structural constituent of muscle	GO:0008307	46	320	7	1.08e-02
MF	integrin binding	GO:0005178	105	367	10	2.70e-02
MF	oxygen transporter activity	GO:0005344	14	239	4	3.18e-02

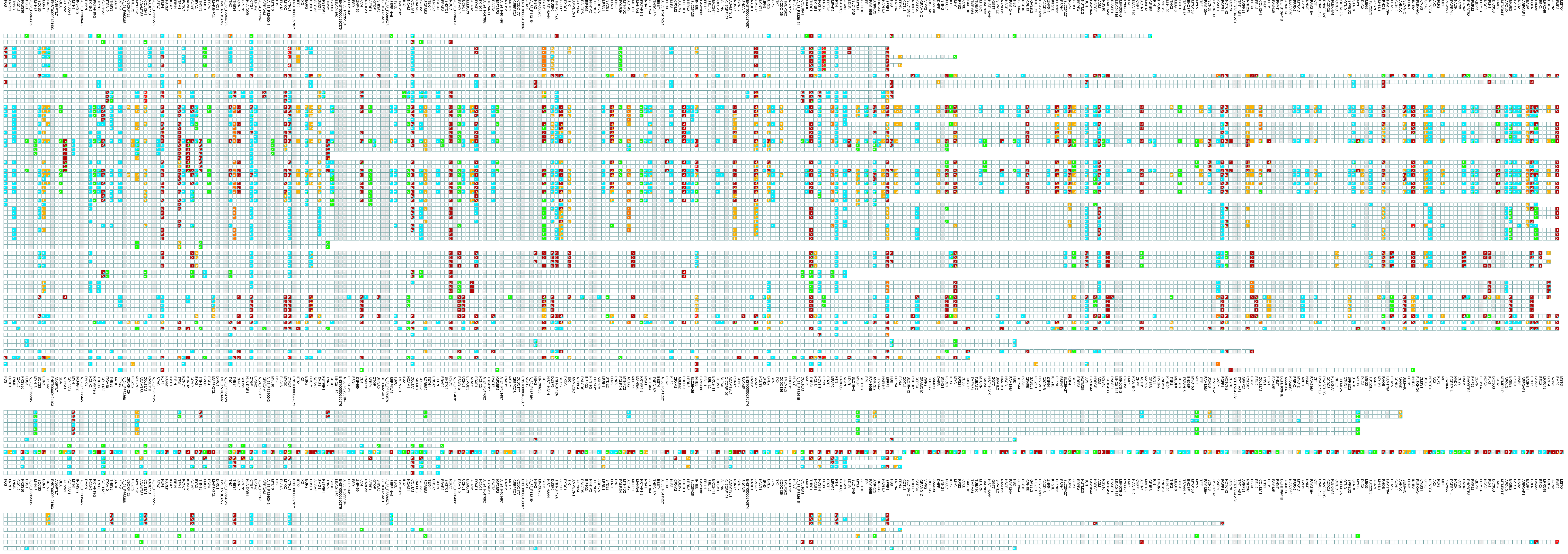

Supplementary Data Set 2.c

p7322 and p2638 (female child, CCD) upregulated genes

source	term name<br>Gene Ontology (Biological process)	term ID	n. of term genes	n. of query genes	n. of common genes	corrected p-value
BP	steroid biosynthetic process	GO:0006694	183	439	15	2.37e-02
BP	bicarbonate transport	GO:0015701	44	16	3	1.34e-02
BP	regulation of dendritic spine development	GO:0060998	64	417	9	1.49e-02
BP	response to oxygen-containing compound	GO:1901700	1512	84	20	7.24e-03
BP	response to alcohol	GO:0097305	200	32	5	4.49e-02
BP	response to ethanol	GO:0045471	139	32	5	7.61e-03
BP	regulation of lipid metabolic process	GO:0019216	358	28	6	2.26e-02
BP	chemical homeostasis	GO:0048878	1052	318	38	1.32e-03
BP	alpha-amino acid metabolic process	GO:1901605	219	425	17	7.89e-03
BP	single-organism process	GO:0044699	13430	141	116	1.23e-03
BP	single-multicellular organism process	GO:0044707	6182	361	143	1.97e-02
BP	single-organism cellular process	GO:0044763	10817	425	271	5.51e-05
BP	cell death	GO:0008219	2075	44	18	3.46e-04
BP	programmed cell death	GO:0012501	1963	44	16	5.59e-03
BP	apoptotic process	GO:0006915	1840	44	16	2.35e-03
BP	cell projection organization	GO:0030030	1364	387	48	3.14e-02
BP	plasma membrane bounded cell projection organization	GO:0120036	1336	387	48	1.81e-02
BP	regulation of biological quality	GO:0065008	3484	312	80	4.37e-02
BP	regulation of hormone levels	GO:0010817	489	412	28	1.41e-03
BP	anatomical structure development	GO:0048856	5547	404	144	4.43e-02
BP	single-organism localization	GO:1902578	3285	425	104	2.67e-03
BP	single-organism metabolic process	GO:0044710	4320	441	127	4.64e-02
BP	small molecule metabolic process	GO:0044281	2128	439	73	4.01e-02
BP	small molecule biosynthetic process	GO:0044283	551	416	29	6.02e-03
BP	organic acid metabolic process	GO:0006082	1123	425	47	5.57e-03
BP	oxoacid metabolic process	GO:0043436	1106	425	47	3.66e-03
BP	carboxylic acid metabolic process	GO:0019752	996	450	45	6.34e-03
BP	oxidation-reduction process	GO:0055114	1001	421	42	1.51e-02
BP	transport	GO:0006810	5035	413	137	2.35e-02
BP	regulation of transport	GO:0051049	1836	421	69	6.11e-04
BP	regulation of peptide transport	GO:0090087	787	421	37	4.39e-03
BP	single-organism transport	GO:0044765	3140	425	101	1.95e-03
BP	gas transport	GO:0015669	20	143	6	1.18e-05
BP	oxygen transport	GO:0015671	15	143	6	1.57e-06
BP	hormone transport	GO:0009914	314	412	23	2.13e-04
BP	anatomical structure morphogenesis	GO:0009653	2449	304	61	3.05e-02
BP	cellular component morphogenesis	GO:0032989	1018	428	42	3.57e-02
BP	cell part morphogenesis	GO:0032990	618	417	31	7.58e-03
BP	cell projection morphogenesis	GO:0048858	599	417	31	3.96e-03
BP	plasma membrane bounded cell projection morphogenesis	GO:0120039	595	417	31	3.44e-03
BP	negative regulation of developmental process	GO:0051093	866	299	29	4.79e-02
BP	response to axon injury	GO:0048678	65	378	9	7.21e-03
BP	multicellular organism development	GO:0007275	5102	393	132	2.99e-02
BP	cell differentiation	GO:0030154	3880	411	111	2.38e-02
BP	cell development	GO:0048468	1938	417	72	3.54e-04
BP	regulation of cell death	GO:0010941	1536	356	56	8.46e-05
BP	regulation of programmed cell death	GO:0043067	1436	58	16	3.08e-03
BP	regulation of apoptotic process	GO:0042981	1422	58	16	2.70e-03
BP	positive regulation of cell death	GO:0010942	628	172	22	3.85e-05
BP	positive regulation of programmed cell death	GO:0043068	592	40	9	5.80e-03
BP	positive regulation of apoptotic process	GO:0043065	586	40	9	5.33e-03
BP	regulation of establishment of protein localization	GO:0070201	834	421	36	4.12e-02
BP	negative regulation of establishment of protein localization	GO:1904950	208	360	14	4.37e-02
BP	regulation of protein transport	GO:0051223	758	421	35	1.28e-02
BP	secretion by cell	GO:0032940	1404	33	11	2.21e-02
BP	signal release	GO:0023061	423	412	26	9.62e-04
BP	hormone secretion	GO:0046879	302	412	22	4.56e-04
BP	peptide secretion	GO:0002790	532	413	32	6.94e-05
BP	peptide hormone secretion	GO:0030072	245	412	18	5.62e-03
BP	regulation of secretion	GO:0051046	695	413	36	3.94e-04
BP	regulation of peptide secretion	GO:0002791	429	413	27	3.71e-04
BP	regulation of secretion by cell	GO:1903530	637	413	34	4.47e-04
BP	regulation of hormone secretion	GO:0046883	256	412	22	2.36e-05
BP	positive regulation of hormone secretion	GO:0046887	119	411	12	1.22e-02
BP	regulation of peptide hormone secretion	GO:0090276	204	412	18	3.77e-04
BP	neurogenesis	GO:0022008	1487	387	52	1.52e-02
BP	generation of neurons	GO:0048699	1390	417	54	4.80e-03
BP	neuron differentiation	GO:0030182	1266	387	49	1.73e-03
BP	neuron development	GO:0048666	1007	387	41	4.62e-03
BP	neuron projection development	GO:0031175	883	428	42	9.63e-04
BP	axon development	GO:0061564	468	387	28	1.45e-04
BP	neuron projection regeneration	GO:0031102	51	378	9	8.55e-04
BP	axon regeneration	GO:0031103	42	378	7	2.82e-02
BP	neuron projection morphogenesis	GO:0048812	583	417	31	2.23e-03
BP	cell morphogenesis involved in differentiation	GO:0000904	662	428	33	7.16e-03
BP	cell morphogenesis involved in neuron differentiation	GO:0048667	533	417	30	1.03e-03
BP	axonogenesis	GO:0007409	432	411	24	1.54e-02
BP	negative regulation of secretion	GO:0051048	196	360	14	2.22e-02
BP	negative regulation of peptide secretion	GO:0002792	113	412	11	4.46e-02
BP	negative regulation of cell differentiation	GO:0045596	644	277	23	4.28e-02
BP	protein secretion	GO:0009306	500	413	30	2.07e-04
BP	insulin secretion	GO:0030073	205	412	17	2.05e-03
BP	regulation of protein secretion	GO:0050708	402	413	25	1.38e-03
BP	regulation of insulin secretion	GO:0050796	175	412	17	2.10e-04
BP	negative regulation of secretion by cell	GO:1903531	172	412	14	2.51e-02
BP	negative regulation of hormone secretion	GO:0046888	65	412	11	1.59e-04
BP	negative regulation of peptide hormone secretion	GO:0090278	42	265	7	2.56e-03

source	term name<br>Gene Ontology (Cellular component)	term ID	n. of term genes	n. of query genes	n. of common genes	corrected p-value
CC	endocytic vesicle lumen	GO:0071682	19	299	5	1.75e-02
CC	cytoplasm	GO:0005737	11137	457	287	1.29e-02
CC	cytoplasmic part	GO:0044444	9275	434	235	1.46e-02
CC	contractile fiber	GO:0043292	221	432	17	1.10e-02
CC	myofibril	GO:0030016	210	432	17	5.47e-03
CC	haptoglobin-hemoglobin complex	GO:0031838	4	72	3	5.41e-04
CC	hemoglobin complex	GO:0005833	12	143	6	2.94e-07
CC	extracellular region part	GO:0044421	4070	126	45	4.97e-02

source	term name<br>Gene Ontology (Molecular function)	term ID	n. of term genes	n. of query genes	n. of common genes	corrected p-value
MF	haptoglobin binding	GO:0031720	3	14	2	3.58e-03
MF	heme binding	GO:0020037	129	238	9	4.49e-02
MF	oxidoreductase activity	GO:0016491	756	423	35	1.36e-02
MF	oxidoreductase activity, acting on the CH-NH group of donors	GO:0016645	28	391	6	3.42e-02
MF	trivalent inorganic cation transmembrane transporter activity	GO:0072510	2	62	2	2.89e-02
MF	ferric iron transmembrane transporter activity	GO:0015091	2	62	2	2.89e-02
MF	cofactor binding	GO:0048037	266	412	19	4.42e-03
MF	oxygen binding	GO:0019825	48	143	6	3.18e-03
MF	cytoskeletal protein binding	GO:0008092	845	453	47	9.26e-06
MF	actin binding	GO:0003779	401	413	24	4.67e-03
MF	iron ion binding	GO:0005506	159	24	5	2.87e-03
MF	transporter activity	GO:0005215	1330	39	12	1.41e-02
MF	substrate-specific transporter activity	GO:0022892	1134	62	14	1.36e-02
MF	oxygen transporter activity	GO:0005344	14	143	6	9.45e-07

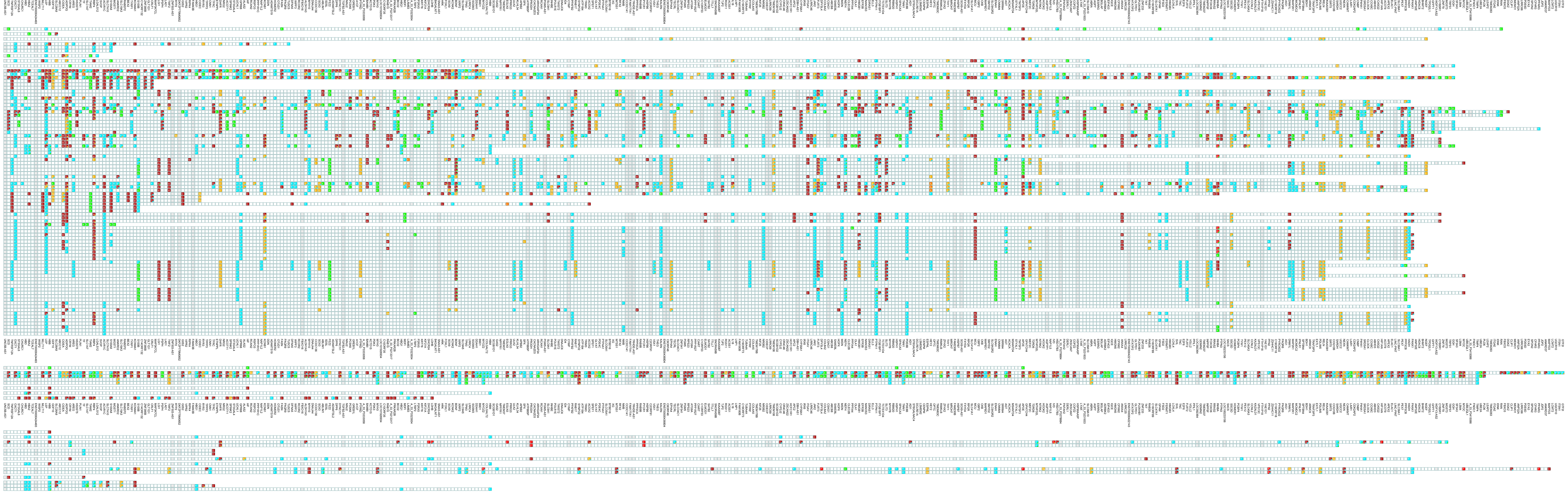

source	term name<br>Biological pathways (KEGG)	term ID	n. of term genes	n. of query genes	n. of common genes	corrected p-value
keg	PPAR signaling pathway	KEGG:03320	72	23	3	2.18e-02
keg	AMPK signaling pathway	KEGG:04152	122	19	3	4.45e-02
keg	African trypanosomiasis	KEGG:05143	34	14	2	3.81e-02

source	term name<br>Biological pathways (Reactome)	term ID	n. of term genes	n. of query genes	n. of common genes	corrected p-value
rea	Fatty acid, triacylglycerol, and ketone body metabolism	REAC:535734	227	412	15	3.13e-02
rea	Metabolism	REAC:1430728	2154	68	22	1.44e-03
rea	O2/CO2 exchange in erythrocytes	REAC:1480926	13	14	2	1.48e-02
rea	Erythrocytes take up oxygen and release carbon dioxide	REAC:1247673	9	14	2	6.84e-03
rea	Erythrocytes take up carbon dioxide and release oxygen	REAC:1237044	13	14	2	1.48e-02

Supplementary Data Set 2.d

p7322 and p2638 (female child, CCD) downregulated genes

source	term name<br>Gene Ontology (Biological process)	term ID	n. of term genes	n. of query genes	n. of common genes	corrected p-value
BP	regulation of heart rate by chemical signal	GO:0003062	6	15	2	2.05e-02
BP	myosin filament organization	GO:0031033	15	61	3	1.93e-02
BP	myosin filament assembly	GO:0031034	14	61	3	1.55e-02
BP	myofibril assembly	GO:0030239	61	17	3	4.61e-02
BP	sarcomere organization	GO:0045214	45	61	4	1.44e-02
BP	striated muscle myosin thick filament assembly	GO:0071688	13	61	3	1.22e-02
BP	multicellular organismal process	GO:0032501	7202	344	157	8.24e-03
BP	system process	GO:0003008	2000	27	16	1.65e-07
BP	muscle system process	GO:0003012	428	27	15	3.97e-16
BP	muscle contraction	GO:0006936	339	25	14	1.72e-16
BP	striated muscle contraction	GO:0006941	167	23	9	1.72e-10
BP	regulation of multicellular organismal process	GO:0051239	2719	347	79	5.37e-04
BP	regulation of system process	GO:0044057	511	322	26	2.34e-04
BP	regulation of muscle system process	GO:0090257	218	27	7	1.81e-05
BP	regulation of muscle contraction	GO:0006937	151	25	6	4.01e-05
BP	developmental process	GO:0032502	5966	335	134	2.98e-03
BP	anatomical structure development	GO:0048856	5547	335	128	1.39e-03
BP	anatomical structure morphogenesis	GO:0009653	2449	293	60	1.35e-02
BP	heart process	GO:0003015	265	17	5	5.17e-03
BP	heart contraction	GO:0060047	261	17	5	4.80e-03
BP	cardiac muscle contraction	GO:0060048	125	17	4	8.15e-03
BP	single-multicellular organism process	GO:0044707	6182	344	145	3.00e-04
BP	multicellular organismal movement	GO:0050879	48	18	4	2.22e-04
BP	musculoskeletal movement	GO:0050881	48	18	4	2.22e-04
BP	skeletal muscle contraction	GO:0003009	38	18	4	8.47e-05
BP	single-organism developmental process	GO:0044767	5877	335	133	2.17e-03
BP	muscle structure development	GO:0061061	609	285	23	2.14e-02
BP	multicellular organism development	GO:0007275	5102	335	120	1.52e-03
BP	system development	GO:0048731	4543	331	106	8.38e-03
BP	animal organ development	GO:0048513	3322	328	83	9.70e-03
BP	muscle organ development	GO:0007517	372	174	14	5.20e-03
BP	circulatory system development	GO:0072359	975	328	37	1.34e-03
BP	actin filament-based process	GO:0030029	676	18	8	1.16e-04
BP	actin filament-based movement	GO:0030048	127	18	8	1.89e-10
BP	actin-mediated cell contraction	GO:0070252	110	18	8	5.81e-11
BP	actin-myosin filament sliding	GO:0033275	39	18	7	4.30e-12
BP	muscle filament sliding	GO:0030049	39	18	7	4.30e-12
BP	oxidoreduction coenzyme metabolic process	GO:0006733	157	288	11	2.67e-02
BP	carbohydrate metabolic process	GO:0005975	576	325	27	8.34e-04
BP	single-organism carbohydrate metabolic process	GO:0044723	455	318	25	7.30e-05
BP	cellular carbohydrate metabolic process	GO:0044262	249	315	18	1.04e-04
BP	regulation of carbohydrate metabolic process	GO:0006109	147	318	12	6.37e-03
BP	regulation of cellular carbohydrate metabolic process	GO:0010675	124	289	10	2.05e-02
BP	pyridine-containing compound metabolic process	GO:0072524	146	288	11	1.33e-02
BP	pyridine nucleotide metabolic process	GO:0019362	141	288	11	9.51e-03
BP	nicotinamide nucleotide metabolic process	GO:0046496	141	288	11	9.51e-03
BP	NAD metabolic process	GO:0019674	73	288	9	1.64e-03
BP	NADH metabolic process	GO:0006734	38	288	8	9.44e-05
BP	NADH regeneration	GO:0006735	27	288	6	3.69e-03
BP	carbohydrate catabolic process	GO:0016052	148	288	15	4.41e-06
BP	single-organism carbohydrate catabolic process	GO:0044724	140	288	15	2.02e-06
BP	glycogen catabolic process	GO:0005980	25	282	5	4.55e-02
BP	monosaccharide metabolic process	GO:0005996	280	289	17	8.20e-04
BP	monosaccharide catabolic process	GO:0046365	62	288	8	4.94e-03
BP	hexose metabolic process	GO:0019318	238	289	17	7.85e-05
BP	hexose catabolic process	GO:0019320	52	288	8	1.23e-03
BP	glucose metabolic process	GO:0006006	196	289	17	4.22e-06
BP	glucose catabolic process	GO:0006007	32	288	7	5.26e-04
BP	pyruvate metabolic process	GO:0006090	105	288	14	3.99e-07
BP	glucose catabolic process to pyruvate	GO:0061718	27	288	6	3.69e-03
BP	gluconeogenesis	GO:0006094	81	289	8	3.87e-02
BP	nucleoside diphosphate metabolic process	GO:0009132	113	288	12	1.16e-04
BP	purine nucleoside diphosphate metabolic process	GO:0009135	92	288	12	1.09e-05
BP	ribonucleoside diphosphate metabolic process	GO:0009185	94	288	12	1.41e-05
BP	purine ribonucleoside diphosphate metabolic process	GO:0009179	92	288	12	1.09e-05
BP	ADP metabolic process	GO:0046031	76	288	12	1.13e-06
BP	nucleotide phosphorylation	GO:0046939	93	288	12	1.24e-05
BP	nucleoside diphosphate phosphorylation	GO:0006165	87	288	12	5.68e-06
BP	ATP generation from ADP	GO:0006757	70	288	11	6.50e-06
BP	glycolytic process	GO:0006096	69	288	11	5.54e-06
BP	glycolytic process through fructose-6-phosphate	GO:0061615	28	288	6	4.65e-03
BP	glycolytic process through glucose-6-phosphate	GO:0061620	28	288	6	4.65e-03
BP	canonical glycolysis	GO:0061621	27	288	6	3.69e-03
BP	response to peptide hormone	GO:0043434	409	289	18	3.56e-02
BP	response to insulin	GO:0032868	253	289	15	5.57e-03
BP	cellular response to insulin stimulus	GO:0032869	197	289	12	4.55e-02
BP	response to inactivity	GO:0014854	15	170	4	1.27e-02
BP	response to muscle inactivity	GO:0014870	12	170	4	4.69e-03
BP	cellular response to ketone	GO:1901655	84	322	9	1.45e-02
BP	cellular response to dexamethasone stimulus	GO:0071549	31	322	6	1.75e-02
BP	cellular response to external stimulus	GO:0071496	278	345	16	4.14e-02
BP	transmembrane receptor protein tyrosine kinase signaling pathway	GO:0007169	690	328	27	3.03e-02
BP	intracellular signal transduction	GO:0035556	2621	347	71	3.33e-02

source	term name<br>Gene Ontology (Cellular component)	term ID	n. of term genes	n. of query genes	n. of common genes	corrected p-value
CC	macromolecular complex	GO:0032991	4869	18	13	4.04e-02
CC	protein complex	GO:0043234	3092	18	11	2.48e-02
CC	supramolecular complex	GO:0099080	901	26	12	2.48e-07
CC	supramolecular polymer	GO:0099081	900	26	12	2.45e-07
CC	supramolecular fiber	GO:0099512	893	18	10	1.81e-06
CC	cytoskeleton	GO:0005856	2032	25	12	1.25e-03
CC	cytoskeletal part	GO:0044430	1536	25	12	5.57e-05
CC	actin cytoskeleton	GO:0015629	449	25	11	1.58e-09
CC	myosin complex	GO:0016459	71	25	7	3.17e-09
CC	myosin filament	GO:0032982	22	12	4	1.77e-06
CC	myosin II complex	GO:0016460	34	25	7	1.31e-11
CC	contractile fiber	GO:0043292	221	25	11	6.44e-13
CC	contractile fiber part	GO:0044449	205	25	11	2.78e-13
CC	muscle myosin complex	GO:0005859	27	25	7	2.18e-12
CC	myofibril	GO:0030016	210	18	9	1.18e-10
CC	myofilament	GO:0036379	19	18	4	4.49e-06
CC	sarcomere	GO:0030017	188	18	8	4.57e-09
CC	A band	GO:0031672	42	17	4	9.82e-05
CC	I band	GO:0031674	138	285	11	6.89e-03
CC	Z disc	GO:0030018	123	164	8	1.20e-02
CC	striated muscle thin filament	GO:0005865	16	18	4	2.11e-06
CC	troponin complex	GO:0005861	8	18	3	8.95e-05

source	term name<br>Gene Ontology (Molecular function)	term ID	n. of term genes	n. of query genes	n. of common genes	corrected p-value
MF	calmodulin binding	GO:0005516	189	291	12	3.24e-02
MF	structural molecule activity conferring elasticity	GO:0097493	17	61	3	2.88e-02
MF	muscle alpha-actinin binding	GO:0051371	18	61	3	3.45e-02
MF	cytoskeletal protein binding	GO:0008092	845	18	9	2.92e-05
MF	actin binding	GO:0003779	401	18	9	4.04e-08
MF	actin filament binding	GO:0051015	144	18	4	1.86e-02
MF	calcium ion binding	GO:0005509	704	25	7	2.54e-02
MF	structural constituent of muscle	GO:0008307	46	25	5	4.48e-06

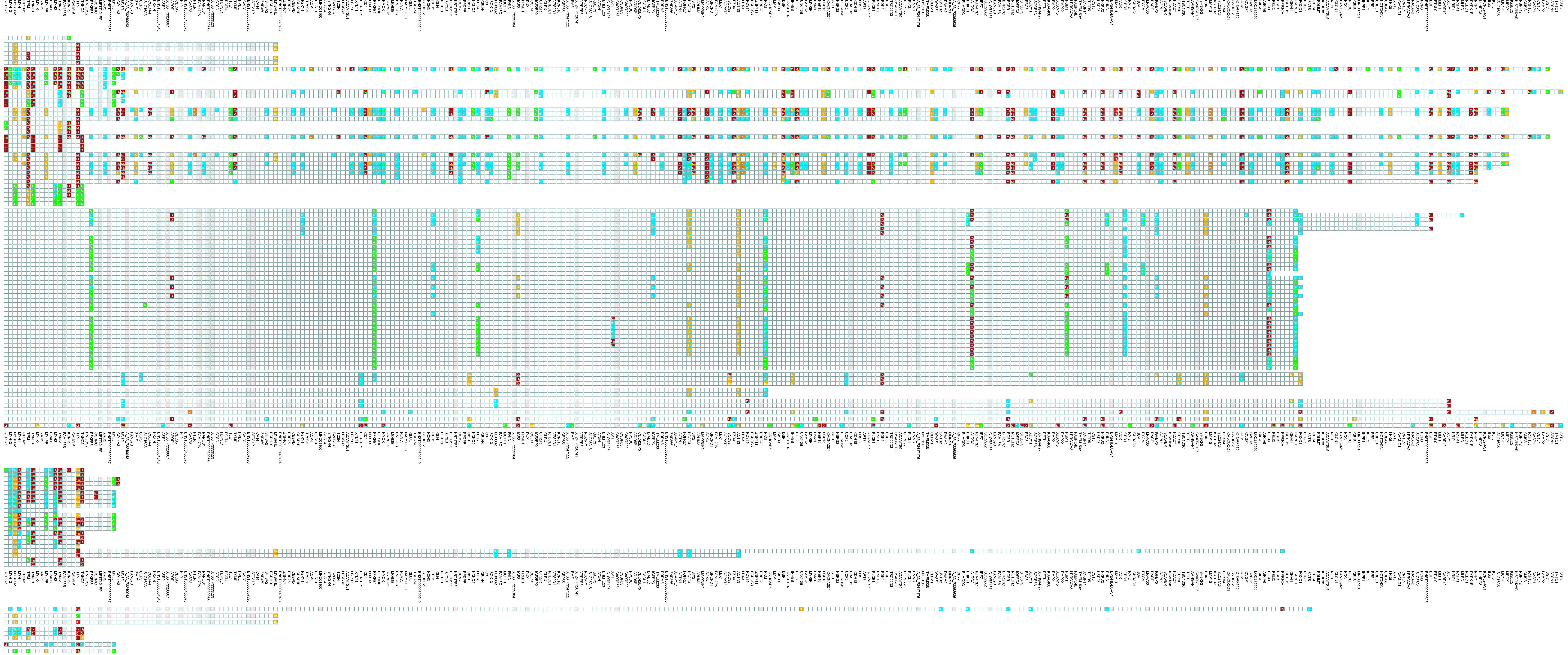

source	term name	term ID	n. of term genes	n. of query genes	n. of common genes	corrected p-value
	**Biological pathways (KEGG)**					
keg	Glucagon signaling pathway	KEGG:04922	103	289	9	2.62e-03
keg	Calcium signaling pathway	KEGG:04020	180	18	4	6.36e-03
keg	HIF-1 signaling pathway	KEGG:04066	101	318	9	4.42e-03
keg	Tight junction	KEGG:04530	170	12	3	3.79e-02
keg	Focal adhesion	KEGG:04510	201	33	5	6.87e-03
keg	Glycolysis / Gluconeogenesis	KEGG:00010	67	288	6	4.94e-02

source	term name	term ID	n. of term genes	n. of query genes	n. of common genes	corrected p-value
	**Biological pathways (Reactome)**					
rea	Metabolism of carbohydrates	REAC:71387	305	315	15	4.70e-02
rea	Glucose metabolism	REAC:70326	83	288	10	1.26e-04
rea	Glycogen breakdown (glycogenolysis)	REAC:70221	18	254	4	2.49e-02
rea	Glycolysis	REAC:70171	34	288	6	3.26e-03
rea	Insulin-like Growth Factor-2 mRNA Binding Proteins (IGF2BPs/IMPs/VICKZs) ...	REAC:428359	8	119	3	3.71e-03
rea	Translocation of GLUT4 to the plasma membrane	REAC:1445148	63	12	3	6.11e-03
rea	Muscle contraction	REAC:397014	200	18	9	6.21e-11
rea	Striated Muscle Contraction	REAC:390522	34	18	6	4.26e-10
rea	Smooth Muscle Contraction	REAC:445355	35	25	3	9.69e-03

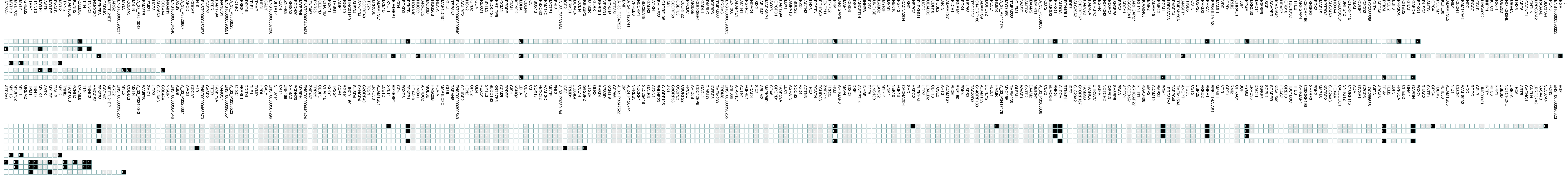

Supplementary Data Set 2.e

p4449 and p7379 (adult female, CCD) upregulated genes

source	term name<br>Gene Ontology (Biological process)	term ID	n. of term genes	n. of query genes	n. of common genes	corrected p-value	A_33_P3422289	XRRA1	A_24_P281395	MMP9	FAM149A	ASB4	ZNF273	AMFR	TSPAN8	CSF3	...
BP	positive regulation of binding	GO:0051099	155	10	3	3.68e-02	?		?	D	?			M		D	
BP	positive regulation of protein binding	GO:0032092	80	10	3	5.02e-03	?		?	D	?			M		D	

source	term name<br>Biological pathways (KEGG)	term ID	n. of term genes	n. of query genes	n. of common genes	corrected p-value	A_33_P3422289	XRRA1	A_24_P281395	MMP9	FAM149A	ASB4	ZNF273	AMFR	TSPAN8	CSF3	...
keg	IL-17 signaling pathway	KEGG:04657	93	10	2	2.43e-02	?	?	?	k	?	?	?		?	k	

Supplementary Data Set 2.f

p4449 and p7379 (adult female, CCD) downregulated genes

source	term name<br>Gene Ontology (Biological process)	term ID	n. of term genes	n. of query genes	n. of common genes	corrected p-value	CRYM	NEXN-AS1	MUC20	LRRC69	LURAP1	TNFRSF12A	LINC00477	VLDLR-AS1	FBXO3	DMRT2	IL12RB2	OLFM1	ST8SIA5	SGK1	MPZL2	ALAS2	CALML6	NUDT4	NRARP	SLC9A3R2	..
BP	regulation of somitogenesis	GO:0014807	9	19	2	4.26e-02		?		?			?	?		e									e		

source	term name<br>Biological pathways (KEGG)	term ID	n. of term genes	n. of query genes	n. of common genes	corrected p-value	CRYM	NEXN-AS1	MUC20	LRRC69	LURAP1	TNFRSF12A	LINC00477	VLDLR-AS1	FBXO3	DMRT2	IL12RB2	OLFM1	ST8SIA5	SGK1	MPZL2	ALAS2	CALML6	NUDT4	NRARP	SLC9A3R2	..
keg	Aldosterone-regulated sodium reabsorption	KEGG:04960	39	20	2	3.06e-02	?	?	?	?	?		?	?	?	?		?		k	?			?	?	k	

Supplementary Data Set 2.g

RyR1$^{I4859T/wt}$ mouse EDL upregulated genes

source	term name<br>Gene Ontology (Biological process)	term ID	n. of term genes	n. of query genes	n. of common genes	corrected p-value
BP	cellular component organization or biogenesis	GO:0071840	5602	348	124	2.51e-02
BP	cellular localization	GO:0051641	2303	361	67	4.96e-03
BP	single-organism cellular localization	GO:1902580	822	337	30	2.61e-02
BP	establishment of localization in cell	GO:0051649	1783	350	53	1.60e-02
BP	intracellular transport	GO:0046907	1492	350	48	5.64e-03
BP	cytosolic transport	GO:0016482	126	337	11	1.00e-02
BP	vacuolar transport	GO:0007034	99	337	10	7.48e-03
BP	lysosomal transport	GO:0007041	75	337	9	5.59e-03
BP	purine nucleoside triphosphate metabolic process	GO:0009144	233	365	15	2.26e-02
BP	ribonucleoside triphosphate metabolic process	GO:0009199	232	365	15	2.15e-02
BP	purine ribonucleoside triphosphate metabolic process	GO:0009205	225	365	15	1.48e-02
BP	tissue development	GO:0009888	1695	330	48	3.31e-02
BP	cellular process	GO:0009987	15804	367	302	1.01e-03
BP	single-organism cellular process	GO:0044763	9764	346	201	1.83e-04
BP	metabolic process	GO:0008152	10902	367	245	1.00e-08
BP	primary metabolic process	GO:0044238	9911	366	219	7.42e-06
BP	organic substance metabolic process	GO:0071704	10312	366	231	1.87e-07
BP	organic cyclic compound metabolic process	GO:1901360	5629	366	140	8.37e-05
BP	catabolic process	GO:0009056	1860	367	67	2.80e-06
BP	organic substance catabolic process	GO:1901575	1566	366	59	5.86e-06
BP	biosynthetic process	GO:0009058	5632	347	133	1.55e-04
BP	organic substance biosynthetic process	GO:1901576	5564	347	133	7.16e-05
BP	macromolecule biosynthetic process	GO:0009059	4685	358	116	9.85e-04
BP	nitrogen compound metabolic process	GO:0006807	9328	366	205	1.05e-04
BP	organonitrogen compound metabolic process	GO:1901564	6205	366	146	1.32e-03
BP	organonitrogen compound catabolic process	GO:1901565	1001	350	38	1.57e-03
BP	cellular metabolic process	GO:0044237	9720	367	227	4.22e-09
BP	cellular nitrogen compound metabolic process	GO:0034641	5973	366	156	1.27e-07
BP	cellular catabolic process	GO:0044248	1565	305	52	6.40e-06
BP	cellular macromolecule metabolic process	GO:0044260	7889	366	176	1.28e-03
BP	cellular aromatic compound metabolic process	GO:0006725	5462	366	133	8.86e-04
BP	heterocycle metabolic process	GO:0046483	5413	366	132	9.32e-04
BP	nucleobase-containing compound metabolic process	GO:0006139	5291	366	130	7.88e-04
BP	cellular biosynthetic process	GO:0044249	5465	366	135	2.77e-04
BP	cellular nitrogen compound biosynthetic process	GO:0044271	4525	365	117	2.50e-04
BP	cellular macromolecule biosynthetic process	GO:0034645	4562	358	115	4.33e-04
BP	developmental process	GO:0032502	5720	348	125	4.33e-02
BP	nucleic acid metabolic process	GO:0090304	4729	357	113	8.00e-03
BP	macromolecule catabolic process	GO:0009057	972	366	37	6.23e-03
BP	cellular macromolecule catabolic process	GO:0044265	794	305	28	1.48e-02
BP	single-organism metabolic process	GO:0044710	3799	366	100	1.71e-03
BP	small molecule metabolic process	GO:0044281	1782	365	58	1.20e-03
BP	positive regulation of biological process	GO:0048518	5180	367	127	1.60e-03
BP	positive regulation of cellular process	GO:0048522	4656	367	112	2.77e-02
BP	single-organism developmental process	GO:0044767	5670	348	124	4.63e-02
BP	head development	GO:0060322	732	125	15	3.05e-02
BP	cellular developmental process	GO:0048869	3994	82	31	1.17e-02
BP	cell differentiation	GO:0030154	3816	82	31	4.33e-03
BP	regulation of metabolic process	GO:0019222	6010	366	137	2.44e-02
BP	regulation of primary metabolic process	GO:0080090	5548	366	128	3.27e-02
BP	regulation of biosynthetic process	GO:0009889	4087	346	101	1.63e-03
BP	regulation of cellular biosynthetic process	GO:0031326	4035	346	97	1.06e-02
BP	positive regulation of metabolic process	GO:0009893	2989	366	86	3.33e-04
BP	positive regulation of nitrogen compound metabolic process	GO:0051173	2662	366	76	3.06e-03
BP	positive regulation of macromolecule metabolic process	GO:0010604	2760	366	77	6.18e-03
BP	positive regulation of biosynthetic process	GO:0009891	1722	270	43	1.24e-02
BP	animal organ development	GO:0048513	3197	348	82	8.68e-03
BP	heart development	GO:0007507	524	329	22	3.63e-02
BP	positive regulation of cellular metabolic process	GO:0031325	2772	366	80	9.38e-04
BP	positive regulation of nucleobase-containing compound metabolic process	GO:0045935	1629	336	47	4.16e-02
BP	positive regulation of cellular biosynthetic process	GO:0031328	1694	270	42	1.94e-02
BP	negative regulation of biological process	GO:0048519	4599	137	49	3.80e-02
BP	gene expression	GO:0010467	5030	358	124	3.76e-04
BP	purine nucleoside monophosphate metabolic process	GO:0009126	242	365	15	3.56e-02
BP	purine ribonucleoside monophosphate metabolic process	GO:0009167	241	365	15	3.39e-02
BP	carboxylic acid metabolic process	GO:0019752	842	343	31	2.23e-02

source	term name<br>Gene Ontology (Cellular component)	term ID	n. of term genes	n. of query genes	n. of common genes	corrected p-value
CC	macromolecular complex	GO:0032991	4878	367	137	3.41e-08
CC	protein complex	GO:0043234	3133	365	89	2.90e-04
CC	catalytic complex	GO:1902494	1293	363	46	1.74e-03
CC	cell	GO:0005623	16677	367	332	2.06e-11
CC	membrane-enclosed lumen	GO:0031974	3859	364	119	1.94e-09
CC	organelle	GO:0043226	12447	367	294	1.24e-19
CC	non-membrane-bounded organelle	GO:0043228	3883	367	115	1.51e-07
CC	membrane-bounded organelle	GO:0043227	11438	367	267	7.55e-14
CC	organelle part	GO:0044422	7720	367	220	7.06e-20
CC	organelle lumen	GO:0043233	3859	364	119	1.94e-09
CC	organelle membrane	GO:0031090	2264	366	69	1.05e-03
CC	cell part	GO:0044464	16636	367	332	1.14e-11
CC	envelope	GO:0031975	1024	366	41	4.51e-04
CC	intracellular	GO:0005622	13545	367	316	5.30e-24
CC	intracellular part	GO:0044424	12955	367	311	1.53e-25
CC	inclusion body	GO:0016234	75	151	6	3.00e-02
CC	cytoplasm	GO:0005737	10019	367	269	2.85e-25
CC	cytoplasmic part	GO:0044444	7725	367	226	1.04e-22
CC	cytosol	GO:0005829	3235	367	112	9.36e-12
CC	intracellular organelle	GO:0043229	11375	367	279	8.47e-20
CC	intracellular non-membrane-bounded organelle	GO:0043232	3883	367	115	1.51e-07
CC	cytoskeleton	GO:0005856	1948	335	59	4.98e-04
CC	microtubule cytoskeleton	GO:0015630	1077	345	38	6.35e-03
CC	contractile fiber	GO:0043292	215	367	18	6.02e-05
CC	contractile fiber part	GO:0044449	194	367	16	4.53e-04
CC	myofibril	GO:0030016	205	367	17	1.71e-04
CC	sarcomere	GO:0030017	183	367	15	1.20e-03
CC	intracellular organelle part	GO:0044446	7516	367	210	5.00e-17
CC	organelle envelope	GO:0031967	1022	366	41	4.28e-04
CC	intracellular organelle lumen	GO:0070013	3856	364	119	1.84e-09
CC	intracellular membrane-bounded organelle	GO:0043231	9787	367	247	2.51e-16
CC	nucleus	GO:0005634	6532	367	162	3.42e-06
CC	nuclear part	GO:0044428	3820	366	108	9.87e-06
CC	nuclear lumen	GO:0031981	3398	351	97	5.16e-06
CC	nucleoplasm	GO:0005654	2618	294	64	1.93e-03
CC	mitochondrion	GO:0005739	1690	365	72	1.55e-10
CC	mitochondrial part	GO:0044429	851	365	44	5.45e-08
CC	mitochondrial envelope	GO:0005740	623	365	33	1.17e-05
CC	mitochondrial matrix	GO:0005759	305	332	19	5.38e-04
CC	mitochondrial membrane	GO:0031966	579	365	29	3.51e-04
CC	organelle inner membrane	GO:0019866	458	365	23	6.98e-03
CC	mitochondrial inner membrane	GO:0005743	404	365	22	3.09e-03
CC	Z disc	GO:0030018	122	367	11	1.68e-02

source	term name<br>Gene Ontology (Molecular function)	term ID	n. of term genes	n. of query genes	n. of common genes	corrected p-value
MF	binding	GO:0005488	13278	367	281	1.14e-08
MF	heterocyclic compound binding	GO:1901363	5919	365	150	3.35e-06
MF	small molecule binding	GO:0036094	2446	346	65	3.28e-02
MF	protein binding	GO:0005515	7962	350	188	1.21e-08
MF	identical protein binding	GO:0042802	1397	349	45	1.09e-02
MF	organic cyclic compound binding	GO:0097159	6011	365	151	5.41e-06
MF	nucleic acid binding	GO:0003676	3952	365	101	5.12e-03
MF	RNA binding	GO:0003723	1593	365	61	1.41e-06
MF	catalytic activity	GO:0003824	5836	349	138	9.73e-05
MF	cadherin binding	GO:0045296	306	361	17	3.46e-02

source	term name<br>Biological pathways (KEGG)	term ID	n. of term genes	n. of query genes	n. of common genes	corrected p-value
keg	Ribosome	KEGG:03010	130	358	10	4.48e-02
keg	Long-term depression	KEGG:04730	61	274	6	2.93e-02
keg	Valine, leucine and isoleucine degradation	KEGG:00280	55	322	9	6.89e-05
keg	Autophagy - other	KEGG:04136	33	335	5	4.02e-02

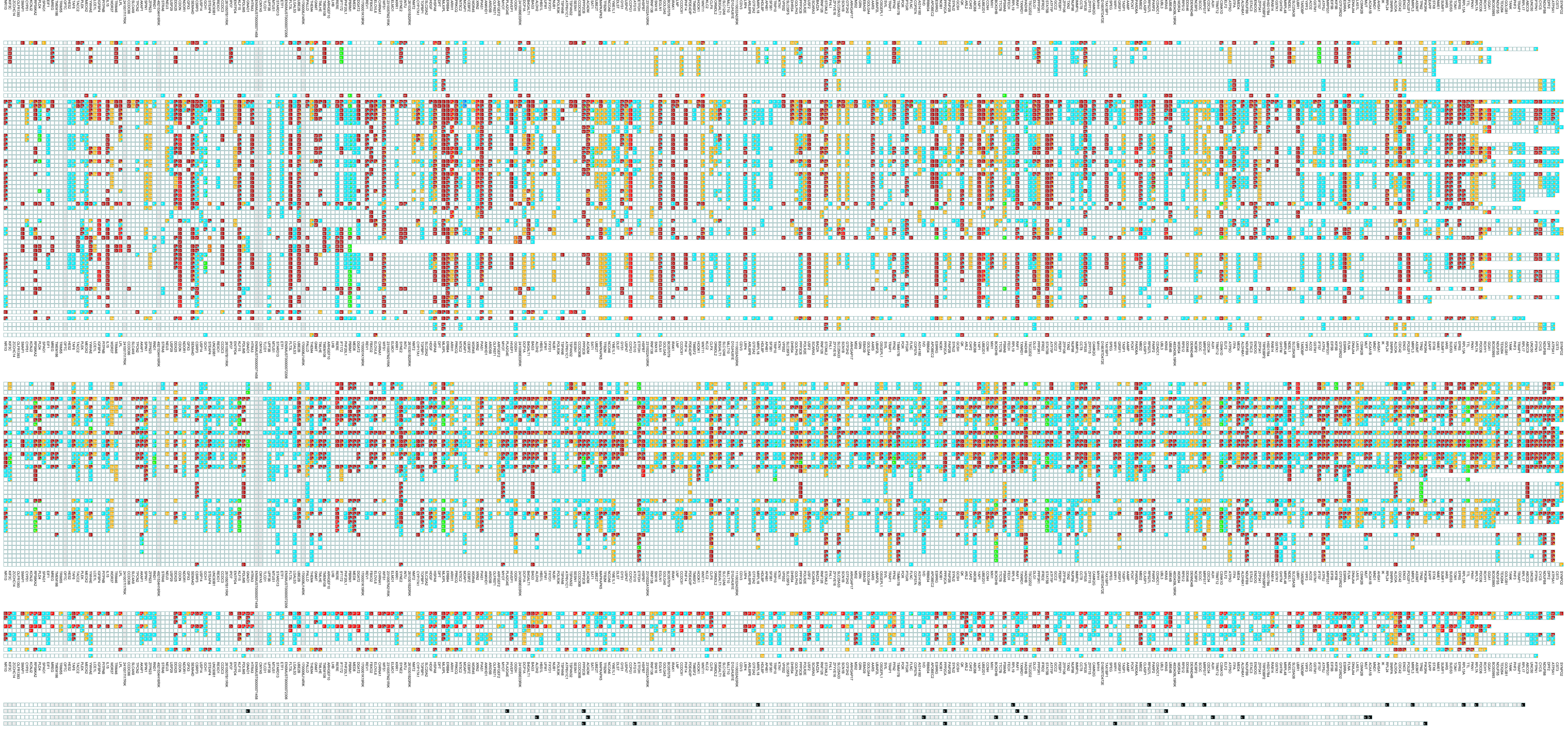

Supplementary Data Set 2.h

RyR1$^{I4859T/wt}$ mouse EDL downregulated genes

source	term name	term ID	n. of term genes	n. of query genes	n. of common genes	corrected p-value
	Gene Ontology (Biological process)					
BP	negative regulation of extrinsic apoptotic signaling pathway via death d ...	GO:1902042	26	265	5	3.90e-02
BP	localization	GO:0051179	5545	350	127	5.29e-03
BP	establishment of localization	GO:0051234	4447	293	94	2.09e-03
BP	transport	GO:0006810	4297	293	89	1.17e-02
BP	vesicle-mediated transport	GO:0016192	1283	210	31	4.00e-03
BP	regulation of vesicle-mediated transport	GO:0060627	421	293	20	2.95e-03
BP	macromolecule localization	GO:0033036	2633	348	69	4.04e-02
BP	protein localization	GO:0008104	2340	348	64	2.34e-02
BP	establishment of protein localization	GO:0045184	1856	341	54	1.33e-02
BP	regulation of localization	GO:0032879	2407	350	69	2.18e-03
BP	amide transport	GO:0042886	1776	341	51	3.66e-02
BP	peptide transport	GO:0015833	1757	341	51	2.73e-02
BP	protein transport	GO:0015031	1717	341	50	3.10e-02
BP	ribonucleoprotein complex subunit organization	GO:0071826	207	29	5	2.27e-02
BP	metabolic process	GO:0008152	10902	348	220	2.43e-04
BP	primary metabolic process	GO:0044238	9911	348	198	9.46e-03
BP	organic substance metabolic process	GO:0071704	10312	348	209	7.29e-04
BP	macromolecule metabolic process	GO:0043170	8780	352	183	4.94e-03
BP	macromolecule modification	GO:0043412	3751	350	96	1.64e-03
BP	nitrogen compound metabolic process	GO:0006807	9328	352	198	1.63e-04
BP	organonitrogen compound metabolic process	GO:1901564	6205	352	145	1.90e-04
BP	protein metabolic process	GO:0019538	5343	352	125	2.99e-03
BP	protein modification process	GO:0036211	3511	350	92	1.09e-03
BP	catabolic process	GO:0009056	1860	189	35	1.69e-02
BP	cellular metabolic process	GO:0044237	9720	348	203	1.03e-04
BP	cellular macromolecule metabolic process	GO:0044260	7889	350	169	2.07e-03
BP	cellular protein metabolic process	GO:0044267	4540	350	112	7.82e-04
BP	cellular protein modification process	GO:0006464	3511	350	92	1.09e-03
BP	protein modification by small protein conjugation or removal	GO:0070647	891	223	25	1.08e-02
BP	protein modification by small protein conjugation	GO:0032446	771	223	22	3.33e-02
BP	cellular catabolic process	GO:0044248	1565	189	33	2.87e-03
BP	macromolecule catabolic process	GO:0009057	972	235	28	4.68e-03
BP	cellular macromolecule catabolic process	GO:0044265	794	235	26	1.06e-03
BP	modification-dependent macromolecule catabolic process	GO:0043632	509	350	23	2.31e-02
BP	protein catabolic process	GO:0030163	763	132	16	3.65e-02
BP	cellular protein catabolic process	GO:0044257	625	350	26	2.57e-02
BP	proteolysis involved in cellular protein catabolic process	GO:0051603	591	350	26	9.46e-03
BP	modification-dependent protein catabolic process	GO:0019941	502	350	23	1.84e-02
BP	ubiquitin-dependent protein catabolic process	GO:0006511	495	350	23	1.47e-02
BP	response to transforming growth factor beta	GO:0071559	192	139	9	1.06e-02
BP	cellular response to transforming growth factor beta stimulus	GO:0071560	191	139	9	1.02e-02
BP	positive regulation of catalytic activity	GO:0043085	1305	210	29	4.55e-02
BP	transcription, DNA-templated	GO:0006351	3452	341	83	4.93e-02
BP	single-organism membrane organization	GO:0044802	642	269	24	3.59e-03
BP	muscle cell apoptotic process	GO:0010657	60	350	8	1.42e-02
BP	cellular component organization or biogenesis	GO:0071840	5602	296	121	6.57e-06
BP	cellular component biogenesis	GO:0044085	2568	139	34	4.50e-02
BP	cellular component organization	GO:0016043	5448	342	132	1.70e-05
BP	organelle organization	GO:0006996	3299	350	88	1.00e-03
BP	single-organism organelle organization	GO:1902589	1455	350	48	3.42e-03
BP	vesicle organization	GO:0016050	260	210	12	2.19e-02

source	term name	term ID	n. of term genes	n. of query genes	n. of common genes	corrected p-value
	Gene Ontology (Cellular component)					
CC	cell-substrate junction	GO:0030055	387	177	13	4.34e-02
CC	cell-substrate adherens junction	GO:0005924	382	177	13	3.79e-02
CC	focal adhesion	GO:0005925	379	177	13	3.48e-02
CC	whole membrane	GO:0098805	1203	138	22	1.47e-02
CC	membrane region	GO:0098589	357	138	11	4.48e-02
CC	organelle	GO:0043226	12447	348	256	2.51e-08
CC	membrane-bounded organelle	GO:0043227	11438	196	144	1.65e-07
CC	vesicle	GO:0031982	3806	154	49	1.70e-02
CC	cell	GO:0005623	16677	348	296	1.20e-02
CC	cell part	GO:0044464	16636	327	278	1.53e-02
CC	intracellular	GO:0005622	13545	348	274	2.38e-09
CC	intracellular part	GO:0044424	12955	320	245	1.03e-08
CC	cytoplasm	GO:0005737	10019	284	181	3.99e-07
CC	cytoplasmic part	GO:0044444	7725	294	151	9.69e-06
CC	cytosol	GO:0005829	3235	319	87	7.72e-06
CC	intracellular organelle	GO:0043229	11375	320	217	3.11e-06
CC	intracellular membrane-bounded organelle	GO:0043231	9787	143	98	4.90e-06
CC	nucleus	GO:0005634	6532	350	141	2.94e-02
CC	organelle part	GO:0044422	7720	138	79	1.11e-04
CC	intracellular organelle part	GO:0044446	7516	138	78	7.83e-05

source	term name	term ID	n. of term genes	n. of query genes	n. of common genes	corrected p-value
	Gene Ontology (Molecular function)					
MF	binding	GO:0005488	13278	352	249	4.32e-02
MF	protein binding	GO:0005515	7962	319	156	3.52e-03
MF	enzyme binding	GO:0019899	1814	341	52	3.11e-02
MF	ion binding	GO:0043167	5894	350	129	4.79e-02
MF	ubiquitin-like protein transferase activity	GO:0019787	430	223	16	2.34e-02
MF	ubiquitin-protein transferase activity	GO:0004842	413	223	16	1.41e-02

source	term name	term ID	n. of term genes	n. of query genes	n. of common genes	corrected p-value
	Biological pathways (KEGG)					
keg	Endocytosis	KEGG:04144	277	153	10	1.51e-02
keg	Apoptosis	KEGG:04210	137	161	8	3.79e-03

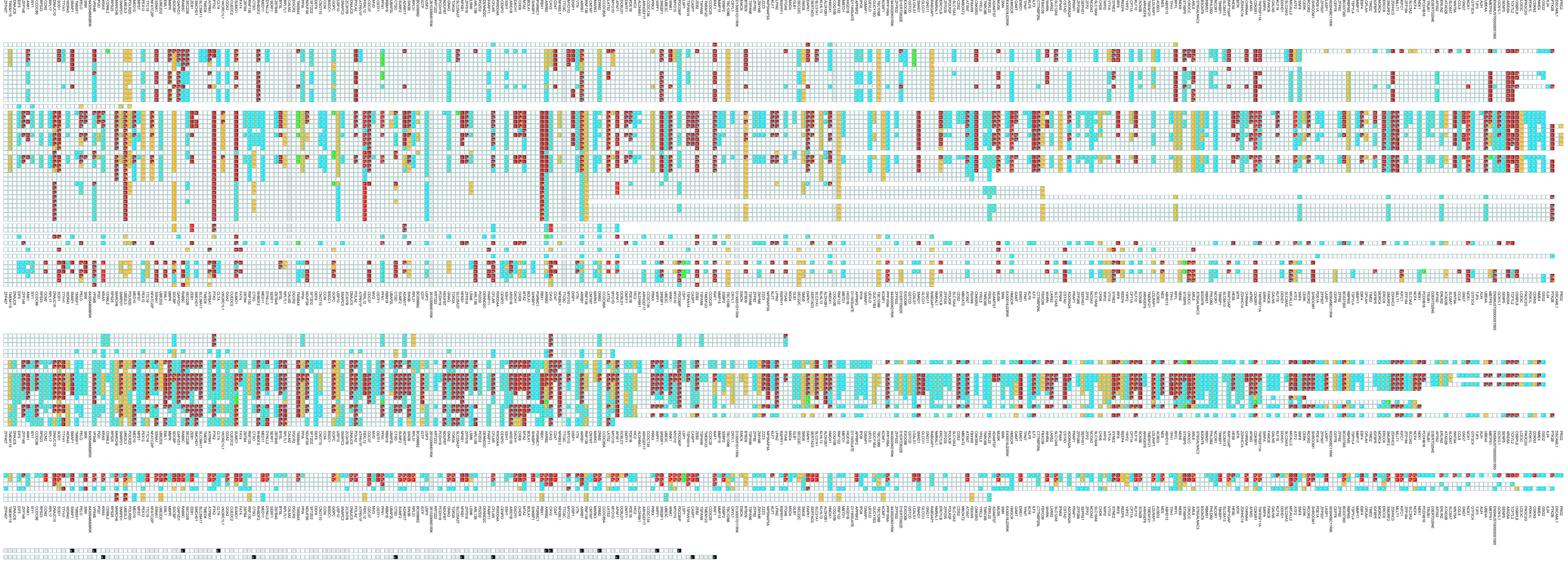

Supplementary Data Set 2.i

RyR1$^{I4859T/wt}$ mouse SOLEUS upregulated genes

source	term name<br>Gene Ontology (Biological process)	term ID	n. of term genes	n. of query genes	n. of common genes	corrected p-value
BP	kidney development	GO:0001822	274	207	12	2.57e-02
BP	metabolic process	GO:0008152	10902	364	219	6.56e-03
BP	cellular metabolic process	GO:0044237	9720	364	197	2.93e-02
BP	cellular hormone metabolic process	GO:0034754	88	8	3	9.76e-03
BP	single-organism metabolic process	GO:0044710	3799	348	96	7.07e-04
BP	small molecule metabolic process	GO:0044281	1782	340	56	2.93e-04
BP	single-organism cellular process	GO:0044763	9764	266	157	1.30e-04
BP	nucleobase-containing small molecule metabolic process	GO:0055086	639	364	27	1.66e-02
BP	nucleoside phosphate metabolic process	GO:0006753	595	364	25	4.06e-02
BP	nucleotide metabolic process	GO:0009117	587	364	25	3.23e-02
BP	isoprenoid metabolic process	GO:0006720	93	8	3	1.15e-02
BP	terpenoid metabolic process	GO:0006721	69	8	3	4.67e-03
BP	terpenoid biosynthetic process	GO:0016114	14	8	2	2.97e-02
BP	diterpenoid metabolic process	GO:0016101	62	8	3	3.38e-03
BP	diterpenoid biosynthetic process	GO:0016102	12	8	2	2.16e-02
BP	retinoid metabolic process	GO:0001523	55	8	3	2.35e-03
BP	retinoic acid metabolic process	GO:0042573	22	8	3	1.38e-04
BP	9-cis-retinoic acid metabolic process	GO:0042905	7	8	2	6.87e-03
BP	retinoic acid biosynthetic process	GO:0002138	12	8	2	2.16e-02
BP	9-cis-retinoic acid biosynthetic process	GO:0042904	7	8	2	6.87e-03
BP	carbohydrate derivative metabolic process	GO:1901135	1066	372	37	4.93e-02
BP	tube morphogenesis	GO:0035239	369	217	14	4.15e-02
BP	organonitrogen compound metabolic process	GO:1901564	6205	366	140	1.14e-02
BP	purine-containing compound metabolic process	GO:0072521	509	364	23	2.85e-02

source	term name<br>Gene Ontology (Cellular component)	term ID	n. of term genes	n. of query genes	n. of common genes	corrected p-value
CC	endosome	GO:0005768	683	261	23	1.21e-02
CC	cell	GO:0005623	16677	373	309	3.12e-02
CC	cell part	GO:0044464	16636	373	309	2.18e-02
CC	envelope	GO:0031975	1024	368	40	9.11e-04
CC	intracellular	GO:0005622	13545	373	274	4.19e-05
CC	intracellular part	GO:0044424	12955	373	262	3.02e-04
CC	cytoplasm	GO:0005737	10019	373	222	4.76e-06
CC	cytoplasmic part	GO:0044444	7725	373	185	1.20e-06
CC	organelle	GO:0043226	12447	373	258	2.72e-05
CC	membrane-bounded organelle	GO:0043227	11438	373	242	3.13e-05
CC	intracellular organelle	GO:0043229	11375	373	231	9.36e-03
CC	intracellular membrane-bounded organelle	GO:0043231	9787	373	209	1.14e-03
CC	mitochondrion	GO:0005739	1690	370	64	6.79e-07
CC	mitochondrial part	GO:0044429	851	368	42	5.26e-07
CC	mitochondrial protein complex	GO:0098798	142	359	12	8.34e-03
CC	organelle envelope	GO:0031967	1022	368	40	8.66e-04
CC	organelle inner membrane	GO:0019866	458	368	23	6.06e-03
CC	mitochondrial envelope	GO:0005740	623	368	29	1.35e-03
CC	mitochondrial matrix	GO:0005759	305	341	17	1.22e-02
CC	mitochondrial membrane	GO:0031966	579	368	27	3.27e-03
CC	mitochondrial membrane part	GO:0044455	195	359	14	8.80e-03
CC	mitochondrial inner membrane	GO:0005743	404	368	22	2.69e-03

source	term name<br>Gene Ontology (Molecular function)	term ID	n. of term genes	n. of query genes	n. of common genes	corrected p-value
MF	oxidoreductase activity, acting on the aldehyde or oxo group of donors	GO:0016903	55	37	4	6.26e-03
MF	ion binding	GO:0043167	5894	369	139	1.59e-03
MF	anion binding	GO:0043168	2668	347	74	7.40e-04
MF	nucleoside phosphate binding	GO:1901265	2118	347	63	6.54e-04
MF	purine ribonucleoside triphosphate binding	GO:0035639	1832	363	59	6.10e-04
MF	carbohydrate derivative binding	GO:0097367	2203	347	65	5.40e-04
MF	small molecule binding	GO:0036094	2446	364	73	3.97e-04
MF	nucleotide binding	GO:0000166	2118	347	63	6.54e-04
MF	ribonucleotide binding	GO:0032553	1889	363	60	7.58e-04
MF	purine nucleotide binding	GO:0017076	1883	363	60	6.80e-04
MF	adenyl nucleotide binding	GO:0030554	1525	363	47	3.90e-02
MF	purine ribonucleotide binding	GO:0032555	1873	363	60	5.67e-04
MF	adenyl ribonucleotide binding	GO:0032559	1516	363	47	3.35e-02
MF	ATP binding	GO:0005524	1485	363	47	1.96e-02
MF	hydrolase activity, acting on acid anhydrides	GO:0016817	839	363	31	4.66e-02
MF	hydrolase activity, acting on acid anhydrides, in phosphorus-containin ...	GO:0016818	837	363	31	4.45e-02
MF	pyrophosphatase activity	GO:0016462	834	363	31	4.15e-02
MF	nucleoside-triphosphatase activity	GO:0017111	795	363	30	4.31e-02
MF	retinal dehydrogenase activity	GO:0001758	8	8	2	9.16e-03
MF	catalytic activity	GO:0003824	5836	366	149	7.07e-07

source	term name<br>Biological pathways (KEGG)	term ID	n. of term genes	n. of query genes	n. of common genes	corrected p-value
keg	Parkinson's disease	KEGG:05012	142	368	11	1.13e-02
keg	Chagas disease (American trypanosomiasis)	KEGG:05142	102	184	6	4.91e-02

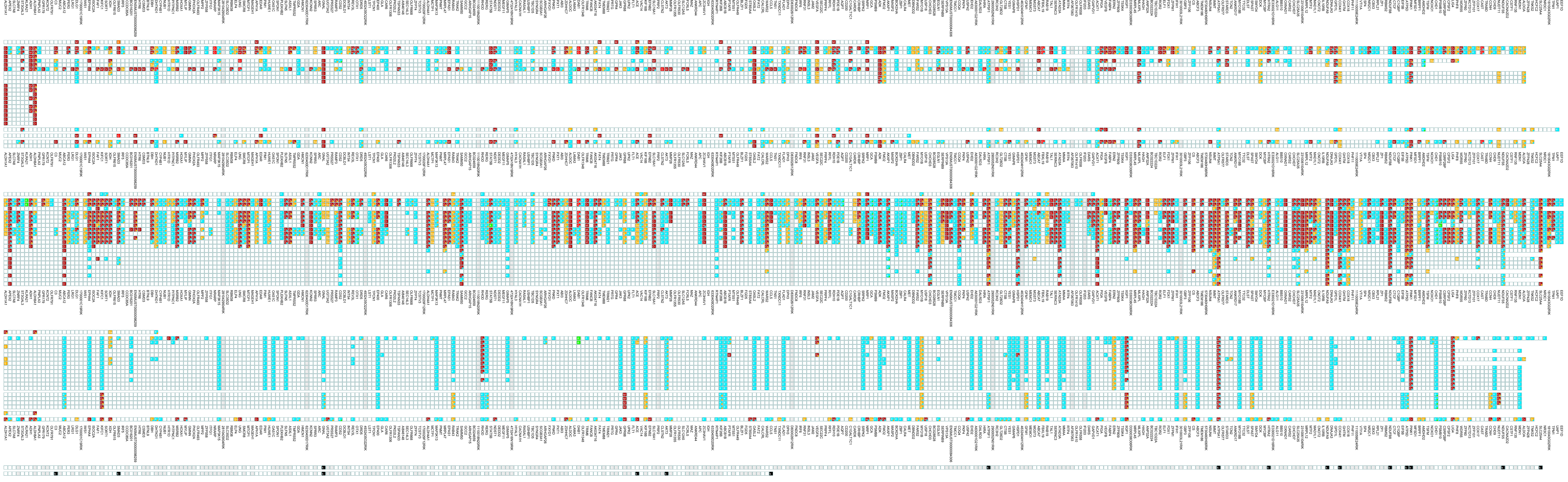

Supplementary Data Set 2.j

RyR1$^{I4859T/wt}$ mouse SOLEUS downregulated genes

source	term name<br>Gene Ontology (Biological process)	term ID	n. of term genes	n. of query genes	n. of common genes	corrected p-value
BP	metabolic process	GO:0008152	10902	234	142	3.57e-02
BP	primary metabolic process	GO:0044238	9911	95	65	4.50e-03
BP	nitrogen compound metabolic process	GO:0006807	9328	95	65	3.14e-04
BP	organic substance metabolic process	GO:0071704	10312	95	66	8.76e-03
BP	macromolecule metabolic process	GO:0043170	8780	95	63	2.14e-04
BP	gene expression	GO:0010467	5030	94	43	1.43e-03
BP	organic cyclic compound metabolic process	GO:1901360	5629	122	52	4.78e-02
BP	cellular metabolic process	GO:0044237	9720	224	127	9.78e-03
BP	cellular macromolecule metabolic process	GO:0044260	7889	95	57	2.04e-03
BP	cellular nitrogen compound metabolic process	GO:0034641	5973	94	47	3.40e-03
BP	nucleobase-containing compound metabolic process	GO:0006139	5291	122	50	4.15e-02
BP	nucleic acid metabolic process	GO:0090304	4729	94	39	1.83e-02
BP	RNA metabolic process	GO:0016070	4246	94	36	2.80e-02
BP	cellular component organization or biogenesis	GO:0071840	5602	289	111	2.24e-04
BP	cellular component organization	GO:0016043	5448	289	108	3.79e-04
BP	regulation of cellular component organization	GO:0051128	2202	275	49	4.59e-02

source	term name<br>Gene Ontology (Cellular component)	term ID	n. of term genes	n. of query genes	n. of common genes	corrected p-value
CC	organelle	GO:0043226	12447	259	190	6.96e-08
CC	membrane-bounded organelle	GO:0043227	11438	140	104	1.79e-05
CC	cell	GO:0005623	16677	319	272	1.32e-05
CC	cell part	GO:0044464	16636	319	272	8.64e-06
CC	endomembrane system	GO:0012505	3477	118	40	2.08e-03
CC	intracellular	GO:0005622	13545	124	108	4.97e-08
CC	intracellular part	GO:0044424	12955	319	235	7.73e-08
CC	cytoplasm	GO:0005737	10019	318	191	3.86e-06
CC	cytoplasmic part	GO:0044444	7725	318	149	1.55e-03
CC	intracellular organelle	GO:0043229	11375	166	122	7.86e-07
CC	intracellular membrane-bounded organelle	GO:0043231	9787	139	91	2.90e-04
CC	Golgi apparatus	GO:0005794	1262	46	12	1.81e-02
CC	organelle part	GO:0044422	7720	238	122	1.56e-05
CC	intracellular organelle part	GO:0044446	7516	238	121	5.65e-06
CC	macromolecular complex	GO:0032991	4878	257	85	1.55e-02

source	term name<br>Gene Ontology (Molecular function)	term ID	n. of term genes	n. of query genes	n. of common genes	corrected p-value
MF	binding	GO:0005488	13278	166	127	1.22e-03
MF	heterocyclic compound binding	GO:1901363	5919	69	36	1.64e-02
MF	protein binding	GO:0005515	7962	302	148	5.92e-04
MF	organic cyclic compound binding	GO:0097159	6011	69	36	2.40e-02
MF	ion channel regulator activity	GO:0099106	80	84	5	3.57e-02

source	term name<br>Biological pathways (KEGG)	term ID	n. of term genes	n. of query genes	n. of common genes	corrected p-value
keg	SNARE interactions in vesicular transport	KEGG:04130	35	9	2	2.43e-02

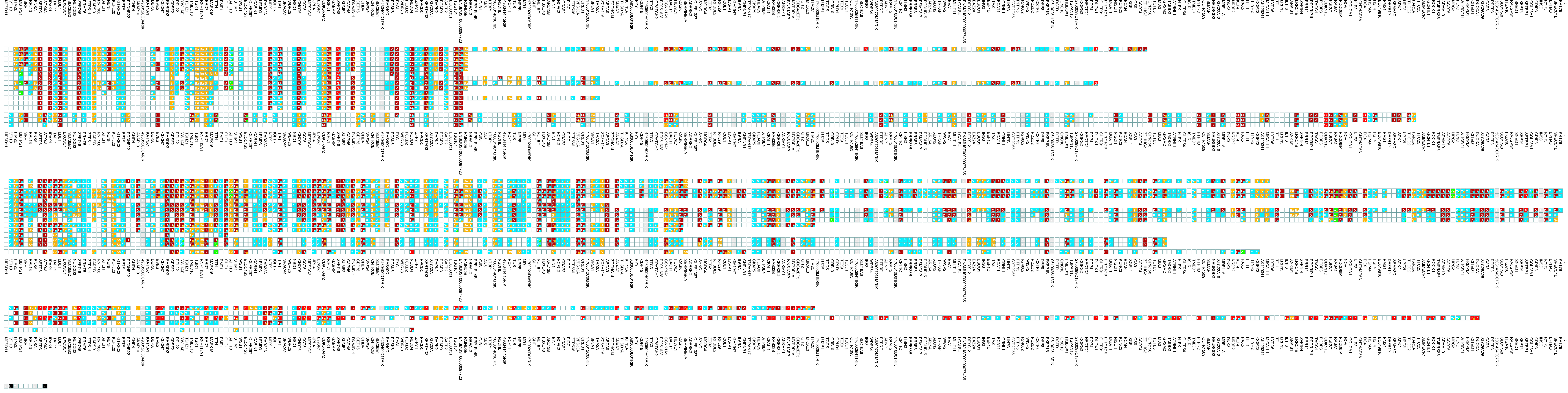

Supplement: Supplementary Data [file ddy149_supp.zip › ddy149-suppl_data/Supplementary Data Set 2.pdf]
